# SGLT2 inhibitor promotes mitochondrial dysfunction and ER-phagy in colorectal cancer cells

**DOI:** 10.1186/s11658-024-00599-1

**Published:** 2024-05-29

**Authors:** Camilla Anastasio, Isabella Donisi, Vitale Del Vecchio, Antonino Colloca, Luigi Mele, Celestino Sardu, Raffaele Marfella, Maria Luisa Balestrieri, Nunzia D’Onofrio

**Affiliations:** 1 Department of Precision Medicine, University of Campania Luigi Vanvitelli, 80138 Naples, Italy; 2 Department of Experimental Medicine, University of Campania Luigi Vanvitelli, Via Luciano Armanni 5, 80138 Naples, Italy; 3 Department of Advanced Clinical and Surgical Sciences, University of Campania Luigi Vanvitelli, 80138 Naples, Italy

**Keywords:** iSGLT2, Metabolic alterations, Mitochondrial dysfunction, SIRT3, ER-stress, Colorectal cancer cells

## Abstract

**Background:**

Sodium-glucose transporter 2 (SGLT2) inhibitors (iSGLT2) are approved medications for type 2 diabetes. Recent studies indicate that iSGLT2 inhibit the growth of some cancer cells. However, the mechanism(s) remains to be fully elucidated.

**Methods:**

The SGLT2 levels were determined in normal colon CCD 841 CoN and, HCT 116, HT-29, SW480 and LoVo colorectal cancer (CRC) cell lines by quantitative real-time PCR and western blot. The effect of iSGLT2 canagliflozin on cell proliferation was examined using CCK-8, as its role on CRC cells metabolism and tumorigenesis has been evaluated by XF HS Seahorse Bioanalyzer and flow cytometric analyses. Transient gene silencing experiments and analysis of protein–protein interaction network were conducted to evaluate the SGLT2 molecular targets in CRC cells.

**Results:**

Data showed that the treatment with iSGLT2 (50 µM) for 72 h induced cell cycle arrest (*p* < 0.001), impaired glucose and energetic metabolism (*p* < 0.001), promoted apoptotic cell death and ER stress flowing into autophagy (*p* < 0.001) in HCT 116 and HT-29 cells. These cellular events were accompanied by sirtuin 3 (SIRT3) upregulation (*p* < 0.01), as also supported by SIRT3 transient silencing experiments resulting in the attenuation of the effects of iSGLT2 on the cellular metabolic/energetic alterations and the induction of programmed cell death. The identification and validation of dipeptidyl peptidase 4 (DPP4) as potential common target of SGLT2 and SIRT3 were also assessed.

**Conclusions:**

These results deepened knowledge on the iSGLT2 contribution in limiting CRC tumorigenesis unveiling the SGLT2/SIRT3 axis in the cytotoxic mechanisms.

**Graphical Abstract:**

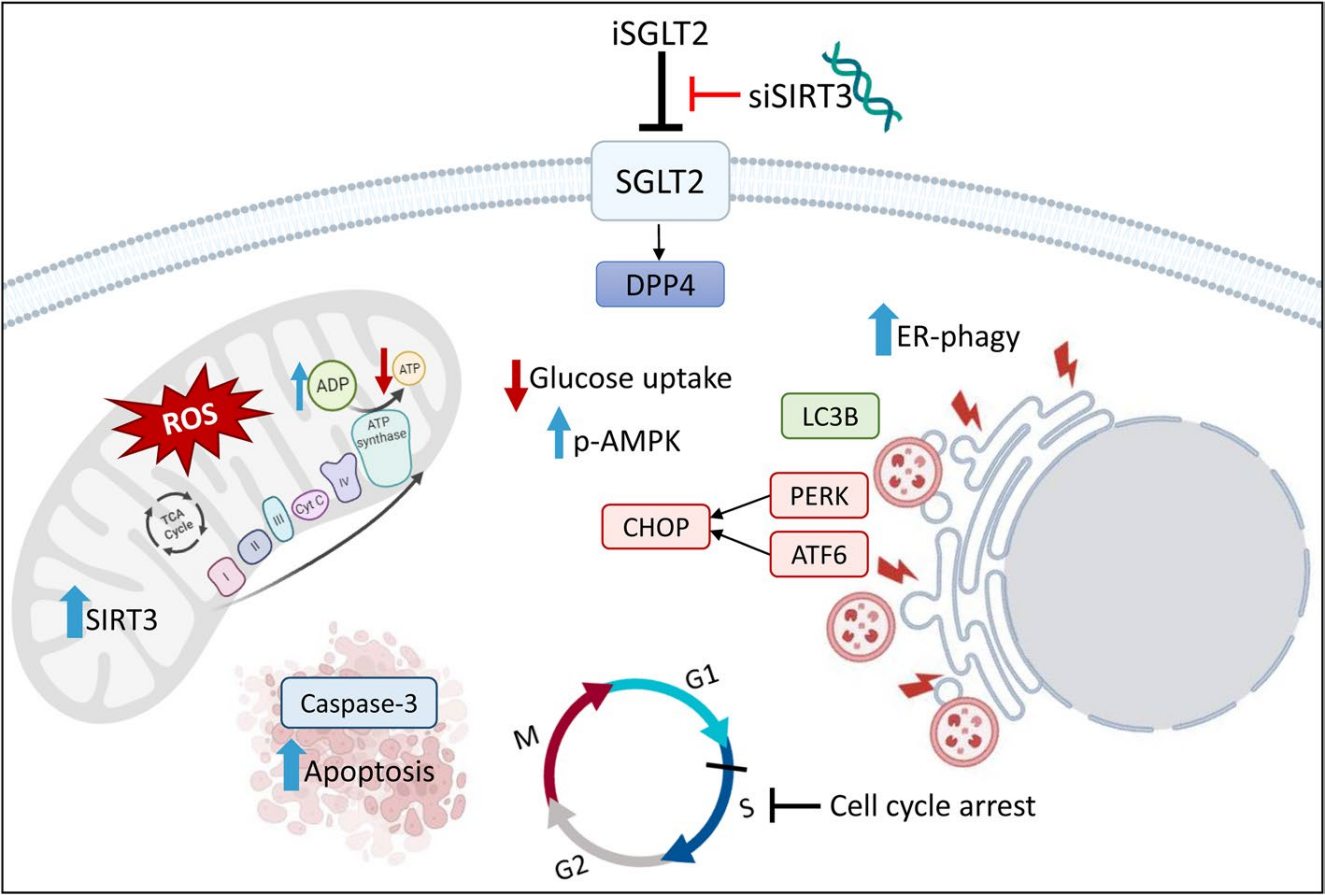

**Supplementary Information:**

The online version contains supplementary material available at 10.1186/s11658-024-00599-1.

## Background

Sodium-glucose cotransporter 2 (SGLT2) is a critical glucose transporter overexpressed in different cancer models, leading to increased glucose uptake in mice and humans [1, 2]. SGLT2 has been found significantly expressed in lung cancer metastasis, pancreatic and prostate adenocarcinomas, high-grade glioblastoma patients, cisplatin-resistant hepatoblastoma, renal cell carcinoma and its increased expression is related to a poor prognosis and decreased overall survival rates [3–7]. Particularly, high glucose levels can directly promote cancer onset and development with different mechanisms, including hyperinsulinemia, inflammation and adipokines imbalance, as well as providing cancer cells with important metabolic substrates [8, 9]. In this scenario, the SGLT2 inhibitors (iSGLT2), canagliflozin, dapagliflozin, tofogliflozin and empagliflozin, have been increasingly studied, given their strong anti-hyperglycemic activity as well as their emerging anticancer effects [8, 10]. These drugs are used to treat patients with type 2 diabetes and chronic kidney disease, as well as to improve cardiac function and reduce the risk of cardiovascular events, including heart failure [11–13]. Treatment with iSGLT2 also results in weight loss, decreased high blood pressure, and ameliorated insulin resistance, lipid profiles, and visceral adiposity [14, 15]. Overall, iSGLT2 are considered pleiotropic drugs exerting multiple beneficial effects, thus being considered as anti-frailty agents [13]. Canagliflozin is an antidiabetic drug acting on proximal renal tubules, able to reduce glucose reabsorption and promote its excretion through the urinary system, without affecting intestinal glucose uptake by SGLT1 [16, 17]. As other iSGLT2, canagliflozin, besides the blood glucose decrease, has been described to have additional beneficial effects, such as reduction of atherosclerotic plaque formation and body weight in rodents and patients with or without type 2 diabetes [14, 18–20]. Growing evidence, in vitro and in vivo, suggested the anticancer effects iSGLT2 canagliflozin, by impairing cell motility and survival as well as exerting cancer specific cytotoxicity alone and combined with oncological drugs [9, 21, 22]. Above all, iSGLT2 could reduce cancer cells glucose uptake and strongly impair cancer specific cell metabolism [9, 23]. Indeed, cancer cells adapt their metabolism to address the requirements of rapid growth, determining oncogenesis and metastasis [24].

Metabolic alterations are particularly critical for the initiation and progression of colorectal cancer (CRC), the third most common neoplasia worldwide, causing 10% of cancer-related deaths in high-income countries and a survival rate from 50 to 93%, depending on the tumor stage [25, 26]. Glucose and redox-energetic pathways differ in CRC cells, since metabolic reprogramming is exploited to provide energy and support for aberrant cell division [27–29], and the enhanced cellular request for glucose can be related to the overexpression of glucose transporters [30]. However, the deepened comprehension of molecular pathways regulating metabolic reprogramming in CRC needs further elucidations. In this context, it is conceivable that SGLT2 modulation in cancer could open a promising field for targeted treatment through iSGLT2. In this context, canagliflozin targets AMP-activated protein kinase (AMPK) activation by complex I of the respiratory chain, inhibits lipid synthesis in mouse liver, promotes mitochondrial remodeling in adipocytes, reduces cell proliferation by impairing mitochondrial respiration in different tumor models, and counteracts renal fibrosis in chronic kidney disease by enhancing sirtuin 3 (SIRT3) expression [31–36]. To date the expression levels, function, and molecular targets of SGLT2 in CRC oncogenesis have not been ascertained. Mechanistically, the knowledge about SGLT2 involvement on glucose homeostasis and mitochondrion-related redox-energetic metabolism in CRC models is still limited, as well as the molecular pathways triggering cytotoxicity. To this end, the present study was designed to investigate the role of SGLT2 on CRC burden and to evaluate the biological effects of its inhibition with iSGLT2, canagliflozin.

## Methods

### Cell culture, treatments, and transfection

Human colon epithelial CCD 841 CoN (CRL-1790) and colorectal adenocarcinoma HCT 116 (CCL-247), HT-29 (HTB-38), SW480 (CCL-228) and LoVo (CCL-229) cells were obtained from American Type Culture Collection (ATCC, Manassas, VA, USA) and grown as previously reported [37]. The iSGLT2 solution, canagliflozin, was prepared as previously described [14]. Briefly, canagliflozin pills were chopped, dissolved in Hanks’ balanced salt solution (HBSS)-10 mM Hepes, homogenized in a Precellys system and centrifugated at 14,000 rpm for 5 min. The supernatant was collected and sterilized through a 0.22 µm Millipore filter. Treatments were performed by culturing cells in complete medium with increasing concentrations of iSGLT2 (0–100 µM) up to 72 h.

As for molecular experiments, cells were transfected with RNAifectin (Vehicle, Applied Biological Materials, Inc., Richmond, BC, Canada) by using 50 nM SIRT3 siRNA (SIRT3^−^, Applied Biological Materials, Inc., Richmond, BC, Canada) or control non-targeting siRNA (NT) in serum- and antibiotic-free medium, as previously reported [38]. After transfection, treatments with iSGLT2 were performed. Control cells (Ctr) were maintained in complete culture medium with the corresponding highest volume of HBSS-10 mM Hepes.

### Cell viability and cytotoxicity

Cell counting Kit-8 and cytotoxicity LDH assays (Donjindo Molecular Technologies, Inc., Rockville, MD, USA) were used to evaluate cell viability and cytotoxicity, respectively, according to manufacturer’s indications. Absorbances were recorded by using the 680-microplate reader (Bio-Rad, Hercules, CA, USA) and results reported as %.

### Cell cycle assessment

After treatment, trypsinized cells were stained with BD Cycletest Plus DNA Kit (BD Biosciences, San José, CA, USA), following manufacturer’s protocol. The intracellular DNA content was analyzed with a FACS CANTO II cytometer (BD Biosciences, San José, CA, USA) and FlowJo V10 software (FlowJo LLC, Ashland, OR, USA). At least 10,000 events were recorded for each sample.

### Metabolic evaluation

Metabolic state was investigated by using Glucose Assay Kit-WST (Dojindo Molecular Technologies, Tokyo, Japan), Lactate Assay Kit-WST (Dojindo, Molecular Technologies, Tokyo, Japan), NAD/NADH Assay Kit-WST (Dojindo Molecular Technologies, Tokyo, Japan), GSSG/GSH quantification kit (Dojindo Molecular Technologies, Tokyo, Japan), and ATP kit (Abcam, Cambridge, UK), according to the supplier’s instructions and as already described [38, 39]. Specific absorbances were measured with a microplate reader model 680 (Bio-Rad, Hercules, CA, USA).

### Oxidative state

Intracellular and mitochondrial ROS levels were detected by CellROX green (Invitrogen, Waltham, MA, USA) and MitoSOX red mitochondrial superoxide indicator (Invitrogen, Waltham, MA, USA), respectively. After treatments, staining was performed with 5 μM of each reagent in complete medium and 30 min incubation at 37 °C. Cells were washed with phosphate buffered saline (PBS) and then imaged on a fluorescence microscope EVOS M5000 (Thermo Scientific, Rockford, USA), while FACS CANTO II (BD Biosciences, San Jose, CA, USA) was used to record fluorescence. Data analyses were performed by FlowJo V10 software (FlowJo LLC, Ashland, OR, USA).

### Apoptotic cell death

Apoptotic mechanism was evaluated with Annexin V apoptosis detection kit (BD Pharmigen, Franklin Lakes, NJ, USA), while caspase-3/7 activation assessed by NucView 488 caspase-3 substrate and annexin V dual apoptosis kit (Biotium, Fremont, CA, USA), as previously reported [38, 40, 41]. Briefly, cells were washed with PBS and incubated for 30 min in binding buffer 1 × containing 2 µL annexin V-FITC and 2 µL propidium iodide (PI) or 5 μL of Annexin V and 5 μL of 0.2 mM NucView 488 caspase-3 substrate for apoptosis and caspase-3/7 detection, respectively. Flow cytometric analyses were performed with FACS CANTO II (BD Biosciences, San Jose, CA, USA) recording at least 10,000 events and results evaluated by FlowJo V10 software (FlowJo LLC, Ashland, OR, USA).

### Mitochondrial respiration

Respiration assay was assessed with the Seahorse MitoStress kit (Agilent Technologies, Santa Clara, CA, USA), as already described [39]. Cells were seeded in Seahorse assay microplate in growth medium or treatment. Culture medium was replaced by complete XF DMEM (Agilent Technologies, Santa Clara, CA, USA) before the detection of oxygen consumption rate (OCR) and extracellular acidification rate (ECAR) conducted by XF HS Seahorse Bioanalyzer (Agilent Technologies, Santa Clara, CA, USA). Results were normalized to protein content.

### SIRT3 activity and content

SIRT3 activity was estimated with SIRT3 fluorometric assay (Enzo Life, Farmingdale, NY, USA), as previously described [38]. Briefly, 50 µL of substrate was added to each well and plate incubated for 5 h, before the addition of 50 µL Developer and 2 mM of nicotinamide (NAM), to stop the reaction. The fluorescence (ex 360 nm and em 460 nm) was measured by Tecan Infinite 2000 Multiplate reader (Tecan, Männedorf, Switzerland). The content of SIRT3 in culture medium was assessed by Sirtuin 3 ELISA Kit (MyBioSource, San Diego, CA, USA), according to the manufacturer’s protocol, and 450 nm absorbance values recorded by a 680-plate reader (Bio-Rad, Hercules, CA, USA).

### Quantitative real-time PCR

RNA isolation and quantitative RT-PCR were performed, as already reported [39], to detect SGLT2 and SIRT3 mRNA levels. The relative sirtuin and SGLT2 expression levels were determined by comparing the expression of SIRT3 or SGLT2 to that of GAPDH with the 2^−ΔΔCt^ method, using the primer sequences reported in Table S1. Each reaction was performed in triplicate and results were expressed as the mean ± SD of *n* = 3 independent experiments.

### ER-phagy detection

Acidic lysosomes and autophagy were detected by LysoTracker Red DND-99 (Invitrogen, Waltham, MA, USA) and Autophagy assay kit (Abcam, Cambridge, UK), respectively. As previously described [39, 42], cells were incubated at 37 °C for 30 min in the dark with 1 µM LysoTracker or 2 μL green reagent and 1 μL nuclear stain. The occurrence of ER stress was assessed by staining cells for 30 min with 1 µM ER-Tracker Blue-White DPX dye (Invitrogen, Waltham, MA, USA). Fluorescent images were captured by EVOS M5000 imaging system (Thermo Scientific, Rockford, USA), while fluorescent signals quantified by FACS CANTO II (BD Biosciences, San Jose, CA, USA). For each sample at least 10,000 events were collected and results analyzed by FlowJo V10 software (FlowJo LLC, Ashland, OR, USA).

### Immunoblotting analysis

Protein lysis, homogenate resolving by sodium dodecyl sulfate–polyacrylamide gel electrophoresis (SDS-PAGE), nitrocellulose membrane transfer, and chemiluminescent analysis were performed as previously described [42, 43]. The following primary antibodies were used for western blotting detection: anti-sodium-glucose cotransporter-2 (SGLT2, 1:500, Abcam, Cambridge, UK), anti-sirtuin 3 (SIRT3, 1:500, Cell Signaling Technology, Danvers, MA, USA), anti-dipeptidyl peptidase-4 (DPP4, 1:500, Abcam, Cambridge, UK) anti-5' AMP-activated protein kinase alpha-1 (AMPK, 1:1000, Invitrogen, Waltham, Ma, USA), anti-phospho-AMPK (1:1000, Invitrogen, Waltham, Ma, USA), anti-C/EBP homologous protein (CHOP, 1:1000, Elabscience Biotechnology Inc., Houston, TX, USA), anti-protein kinase RNA-like ER kinase (PERK, 1:1000, Elabscience Biotechnology Inc., Houston, TX, USA), anti-activating transcription factor 6 (ATF6, 1:1000, Biorbyt, Cambridge, UK), anti-microtubule-associated protein 1 light chain 3B (LC3B, 1:1000, Abcam, Cambridge, UK), anti-actin (1:5000, Abcam, Cambridge, UK), and anti-α-tubulin (1:3000, Elabscience Biotechnology Inc., Houston, TX, USA).

### Analysis of protein–protein interaction network

SGLT2 and SIRT3 interactions were firstly detected by UniProtKB (https://www.uniprot.org/uniprotkb/P31639/entry#interaction) and networks visualized by retrieving direct interactors from the String database version 11.5 (https://version-11-5.string-db.org/cgi/network?taskId=bsoCJQUptkCT&sessionId=b1DrF8N2TVND). The minimum required interaction score value between DDP4 (ENSP00000353731) and SGLT2 (ENSP00000327943) was 0.910, corresponding to highest confidence, in human species. Putative homologs mentioned together in other organisms display 0.043 as score value. Of contrary, none DDP4-SIRT3 network relationship was predicted by the interaction database.

### Statistical analysis

Results were represented as mean ± SD. Graphs and statistical analysis, as Student *t*-test and one-way ANOVA with Tukey post hoc test, were performed using GraphPad Prism version 9.1.2 and differences considered statistically significant as *p* < 0.05.

## Results

### SGLT2 levels in CRC cells and iSGLT2-induced cytotoxicity

**Fig. 1:**
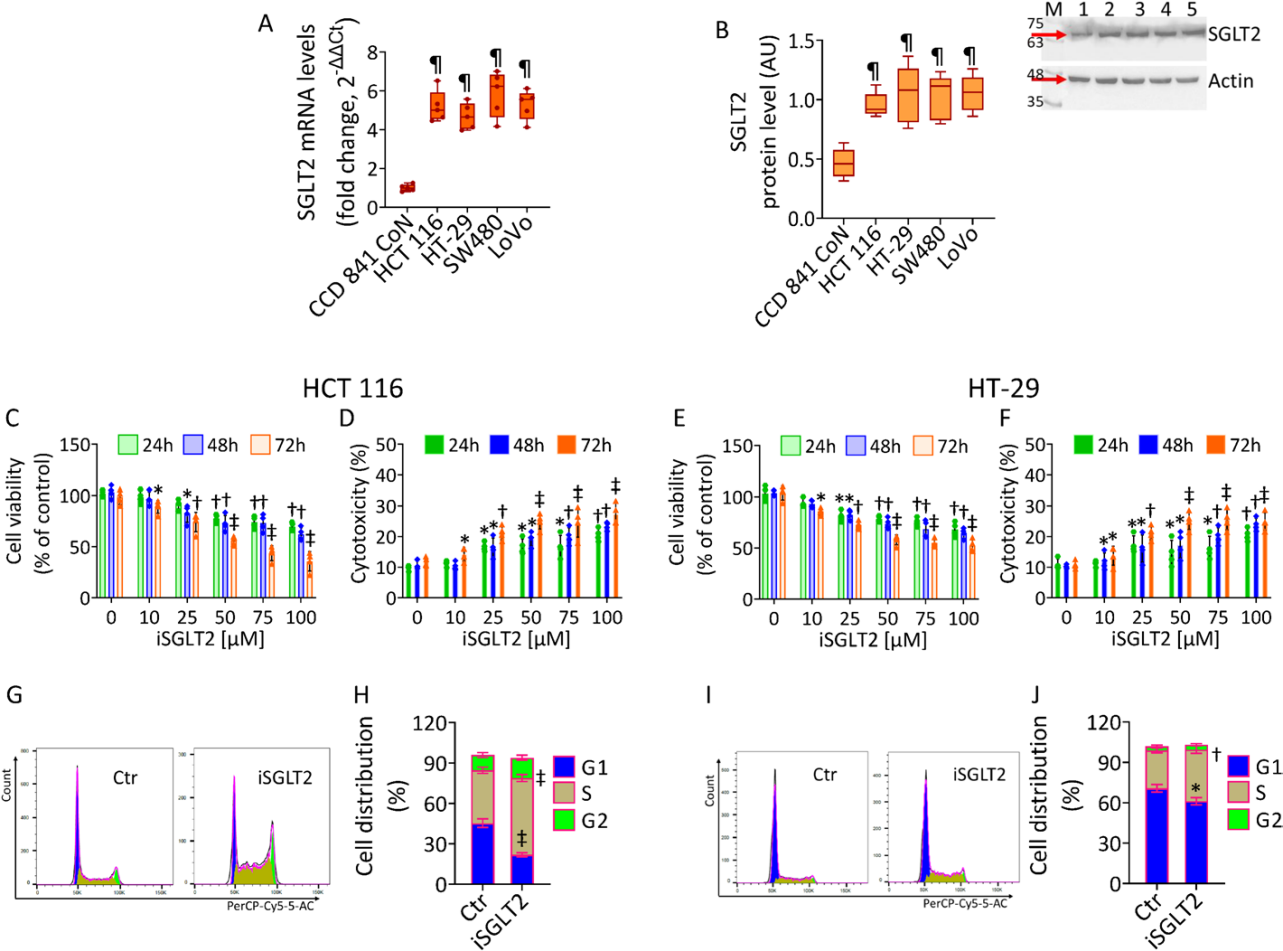
SGLT2 levels and iSGLT2-mediated cytotoxicity in CRC. Basal SGLT2 (**A**) mRNA expression, measured by qRT-PCR, and (**B**) protein levels, detected by immunoblotting, in CCD 841 CoN, HCT 116, HT-29, SW480, and LoVo cells. M = molecular weight markers, lane 1 = CCD 841 CoN, lane 2 = HCT 116, lane 3 = HT-29, lane 4 = SW480, lane 5 = LoVo. Western blotting is expressed as arbitrary units (AU), mRNA levels are reported as floating bars with a line representing the median ± SD of *n* = 3 independent experiments. ¶*p* < 0.01 vs. CCD 841 CoN. Cell viability and cytotoxicity evaluated in (**C**, **D**) HCT 116 and (**E**, **F**) HT-29 cells treated with iSGLT2 (0–100 µM) for 24, 48 and 72 h. Representative cell cycle detection by cytometric analysis in (**G**, **H**) HCT 116 and (**I**, **J**) HT-29 cells exposed to 50 µM iSGLT2 for 72 h (iSGLT2). Control cells (0 or Ctr) were maintained in complete culture medium with the corresponding highest volume of HBSS-10 mM Hepes. The data are expressed as the mean ± SD of *n* = 3 independent experiments. *p < 0.05 vs. 0 µM or Ctr; †*p* < 0.01 vs. 0 µM or Ctr; ‡*p* < 0.001 vs. 0 µM or Ctr by unpaired Student’s *t*-test

To investigate the role of glucose transporter SGLT2 in the metabolic pathways and tumorigenesis of CRC cells, firstly the SGLT2 mRNA and protein levels were determined in normal colon CCD 841 CoN and four CRC cell lines, HCT 116, HT-29, SW480 and LoVo (Fig. 1). The qRT-PCR analysis revealed that the expression levels of SGLT2 were higher in CRC cells (5.19 ± 0.82—in HCT 116, 4.69 ± 0.67—in HT-29, 5.84 ± 1.18—in SW480 and 5.28 ± 0.77-fold change in LoVo) compared to normal CCD 841 CoN cells (0.99 ± 0.17-fold change, *p* < 0.01) (Fig. 1A). A similar trend was observed for SGLT2 protein levels (*p* < 0.01) (Fig. 1B).

To further evaluate the functional involvement of SGLT2, canagliflozin, the iSGLT2 used in the present study from now on referred to as iSGLT2, was tested on cell viability, by measuring the NADH in living cells and cytotoxicity, by measuring the release of LDH from dead cells (Fig. 1 and Fig. S1). Dose–response experiments indicated that 10 µM and 25 µM iSGLT2 treatment poorly reduced HCT 116 and HT-29 cell viability (88.95 ± 6.31 and 85.78 ± 3.39, *p* < 0.05; 74.09 ± 9.42 and 72.45 ± 3.91, *p* < 0.01), while less pronounced reduction was observed in SW480 and LoVo cell (Fig. 1 and Fig. S1). When treated with 50 µM iSGLT2, SW480 and LoVo cells increased their reduction of cell viability (71.28 ± 9.6 and 78.78 ± 5.5, *p* < 0.01 and *p* < 0.05) (Fig. S1A,C), while 50 µM iSGLT2 was the lowest effective concentration able to impair HCT 116 and HT-29 cell viability about 50% (55.86% ± 4.39 and 57.78% ± 4.98, *p* < 0.001) and induce the highest cytotoxic effects after 72 h of treatment (24.96% ± 2.59 and 25.21% ± 2.94, *p* < 0.001) (Fig. 1C–F), suggesting the higher responsiveness and sensibility of HCT 116 and HT-29 cells to iSGLT2 treatment, compared to SW480 and LoVo cell lines.

Moreover, the incubation with iSGLT2 at the highest dose (100 µM) was able to heavily decrease HCT 116 and HT-29 cell viability (35.7 ± 9.4 and 52.5 ± 6.04, *p* < 0.001) (Fig. 1C, E) and aggravate the SW480 and LoVo cell viability reduction (62.75% ± 6.79 and 70.25% ± 6.91, *p* < 0.01) and cytotoxicity (21.27% ± 4.76 and 22.12% ± 2.36, *p* < 0.01) (Fig. S1A–D), furtherly underlining the higher HCT 116 and HT-29 cell susceptibility to iSGLT2 treatment. In contrast, non-malignant CCD 841 CoN cells were not affected by iSGLT2 treatment (Fig. S1E), suggesting that the SGLT2 inhibitor was specific to tumor cells.

Based on this results, the iSGLT2 concentration of 50 µM was chosen to conduct the experiments of the present study on HCT 116 and HT-29 cells.

When cell cycle analysis was performed, iSGLT2 induced the arrest in S phase arrest in HCT 116 (56.94% ± 2.62 vs. 39.39% ± 2.40 of Ctr, *p* < 0.001) and HT-29 cells (38.19% ± 2.84 vs. 28.06% ± 1.90 of Ctr, *p* < 0.01) (Fig. 1G–J).

In conclusion, treatment with iSGLT2 was able to specifically decrease CRC cell viability, induce cytotoxicity and promote cell cycle arrest in HCT 116 and HT-29 cells.

### iSGLT2 effects on glucose metabolism

**Fig. 2:**
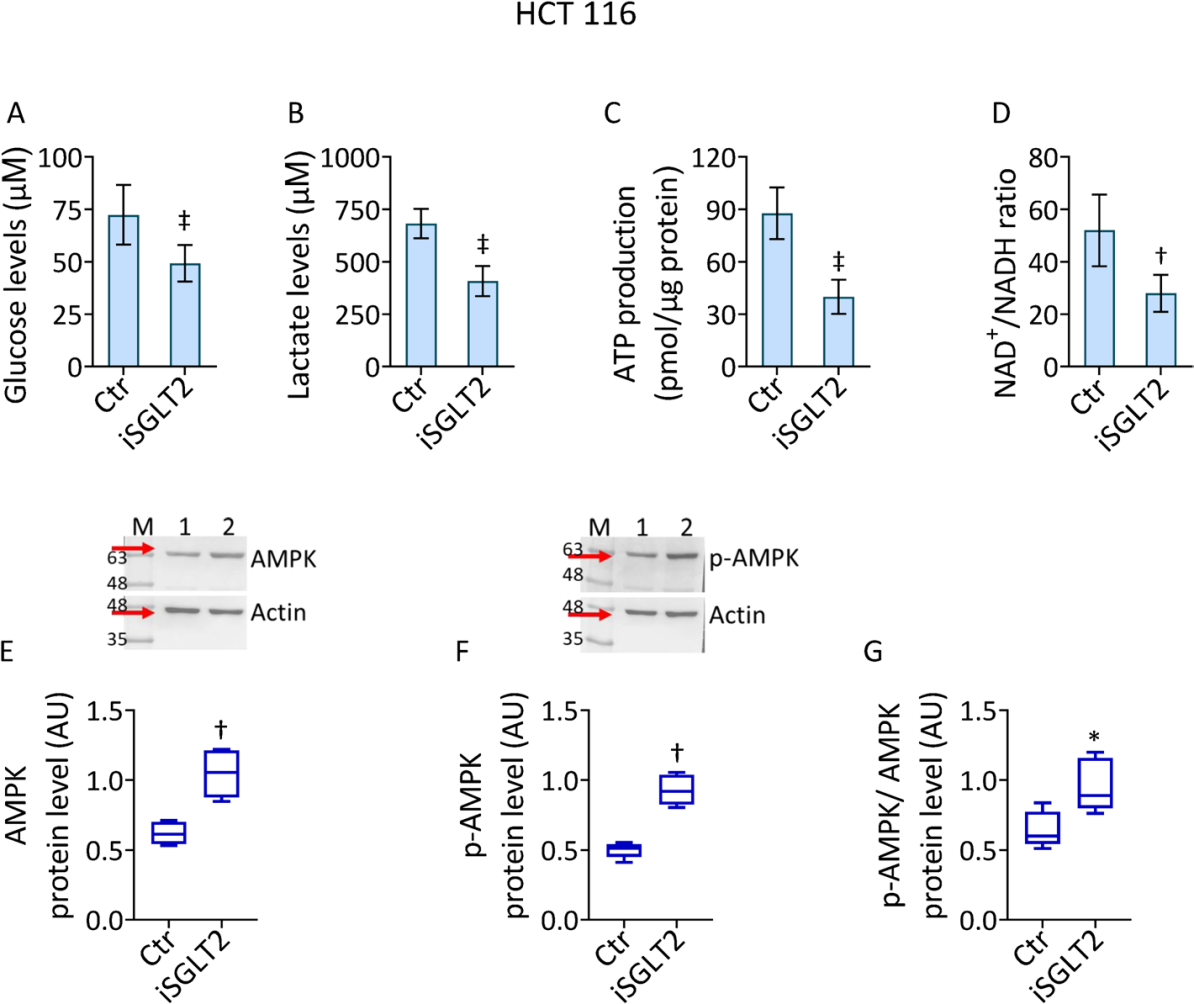
iSGLT2 affects glucose metabolism in CRC. Evaluation of (**A**) glucose, (**B**) lactate, (**C**) ATP and (**D**) NAD^+^/NADH levels and immunoblotting analysis with cropped blots of (**E**) AMPK, (**F**) *p*-AMPK and (**G**) *p*-AMPK/AMPK ratio in HCT 116 cells treated with 50 µM iSGLT2 for 72 h (iSGLT2). Control cells (Ctr) were maintained in complete culture medium with the corresponding volume of HBSS-10 mM Hepes. M = molecular weight markers, lane 1 = Ctr, lane 2 = iSGLT2. Western blotting results are expressed as arbitrary units (AU). *p < 0.05 vs. Ctr; †*p* < 0.01 vs. Ctr; ‡*p* < 0.001 vs. Ctr, by unpaired Student’s *t*-test

The effects of SGLT2 inhibition on glucose metabolism was then evaluated (Fig. 2 and Fig. S2). Exposure to iSGLT2 resulted in decreased glucose and lactate content, as well as ATP production and NAD^+^/NADH ratio in HCT 116 cells (*p* < 0.001) (Fig. 2A–D). These effects were accompanied by increased AMPK protein expression (*p* < 0.01), as evidenced by the evaluation of its total and phosphorylated levels and by phosphorylated/total ratio in iSGLT2-treated cells (Fig. 2E–G). Similarly, in HT-29 cells, treatment with iSGLT2 impaired glucose, lactate ATP levels and NAD^+^/NADH ratio (*p* < 0.01), while enhancing AMPK expression (*p* < 0.05) (Fig. S2).

Overall, iSGLT2 treatment affected glucose metabolism, by decreasing glucose and lactate content, ATP production, NAD + /NADH ratio and increasing AMPK protein expression in CRC cells.

### iSGLT2 causes mitochondrion-mediated oxidative stress and apoptosis

**Fig. 3:**
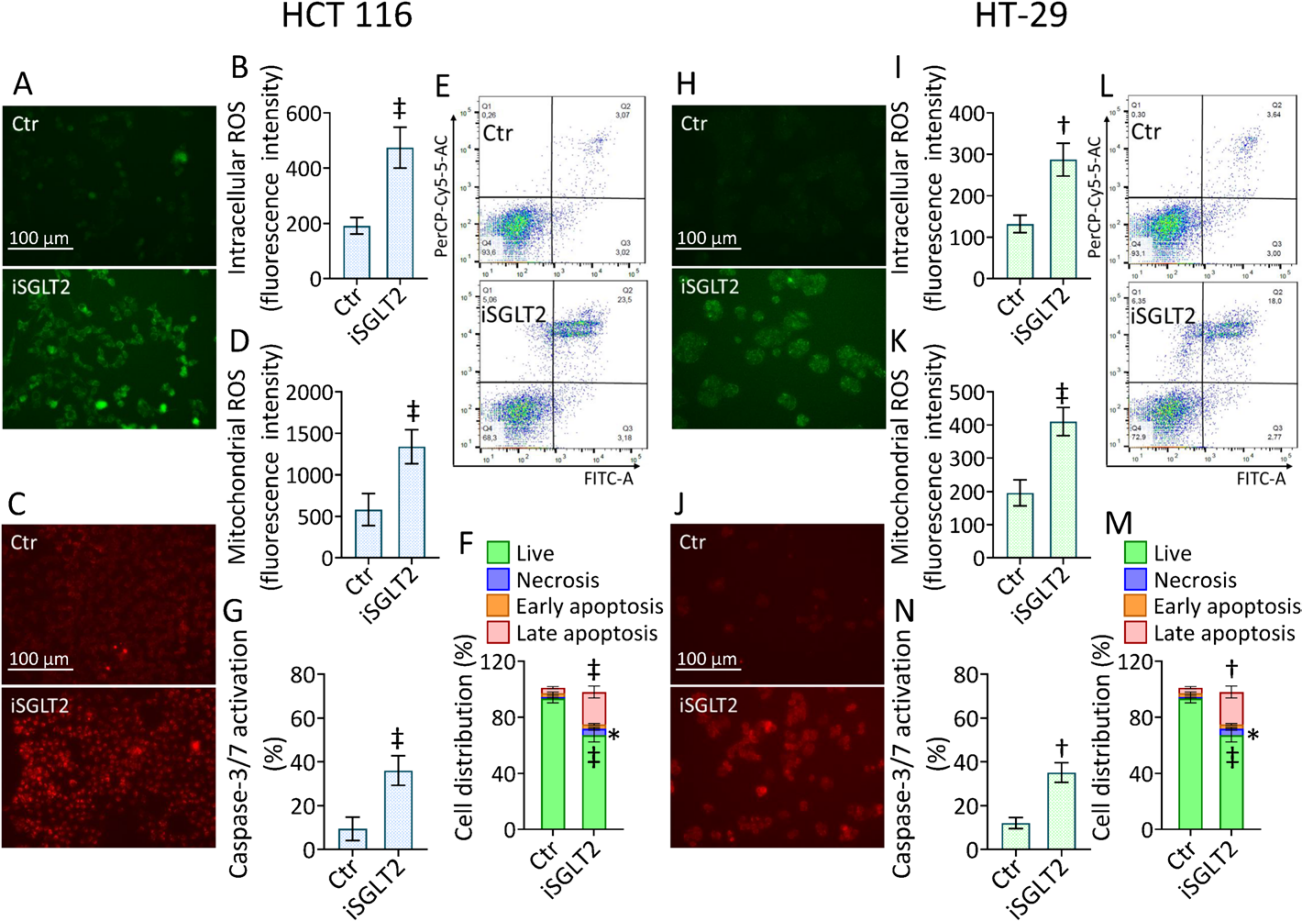
iSGLT2 causes mitochondrion-mediated oxidative stress and apoptosis in CRC. Representative fluorescent images and cytofluorimetric detection of (**A**, **B** and **H**, **I**) intracellular and (**C**, **D** and **J**, **K**) mitochondrial ROS levels, and dot plots and analyses of (**E**, **F** and **L**, **M**) annexin V-FITC and propidium iodide (PI)-staining and (**G**, **N**) detection of caspase-3/7 activation in HCT 116 and HT-29 cells treated with 50 µM iSGLT2 for 72 h (iSGLT2). Control cells (Ctr) were maintained in complete culture medium with the corresponding volume of HBSS-10 mM Hepes. Scale bars = 100 µm. Lower left quadrant: viable cells; upper left quadrant: necrotic cells; lower right quadrant: early apoptotic cells; upper right quadrant: late apoptotic cells. *p < 0.05 vs. Ctr; †*p* < 0.01 vs. Ctr; ‡*p* < 0.001 vs. Ctr, by unpaired Student’s *t*-test

To investigate the SGLT2 inhibition-mediated cytotoxicity, oxidative state and apoptotic cell death were explored under iSGLT2 exposure (Fig. 3 and Fig. S3). The content of intracellular and mitochondrial reactive oxygen species (ROS) during iSGLT2 treatment showed an extensive accrual of reactive species in both cellular compartments of HCT 116 cells (*p* < 0.001) (Fig. 3A–D and Fig. S3A, B). Exposure to iSGLT2 decreased live cells (68.3% ± 4.72 vs. 93.6% ± 3.02 in Ctr, *p* < 0.001), induced late apoptotic accumulation (23.5% ± 4.20 vs. 3.07% ± 0.84 in Ctr, *p* < 0.001) (Fig. 3E, F) and caspase-3 activation (*p* < 0.001) (Fig. 3G and Fig. S3C). Likewise, iSGLT2 mediated intracellular and mitochondrial ROS generation (*p* < 0.001), live cell reduction (72.9% ± 3.31 vs. 93.1% ± 2.91 in Ctr, *p* < 0.001) with late apoptotic rate increase (18.0% ± 1.94 vs. 3.94% ± 0.84 in Ctr, *p* < 0.01) and caspase-3 activation (*p* < 0.01) in HT-29 cells (Fig. 3H–N and Fig. S3D–F).

Comprehensively, iSGLT2 treatment promoted oxidative stress and induced apoptosis of CRC cells via caspase 3 activation.

### iSGLT2 impairs energetic state and modulates SIRT3

**Fig. 4:**
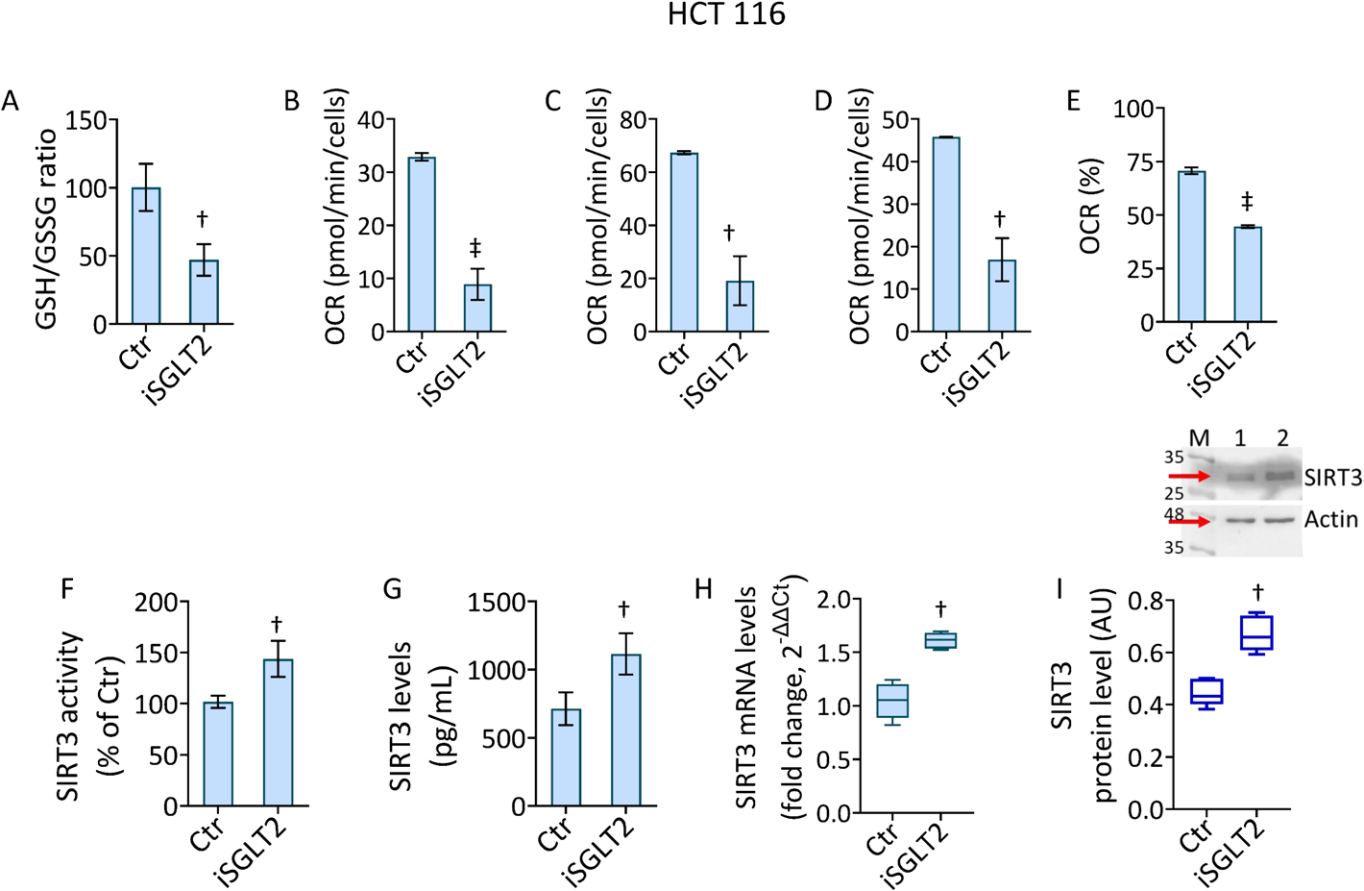
iSGLT2 impairs energetic state and modulates SIRT3 levels in CRC. Evaluation of (**A**) GSH/GSSG ratio and (**B**) ATP production coupled respiration, (**C**) maximal and (**D**) basal respiration, and (**E**) coupling efficiency assessed by Seahorse analyzer and (**F**) SIRT3 activity, (**G**) content evaluated by ELISA kit, (**H**) mRNA expression and (**I**) immunoblotting analysis with cropped blots in HCT 116 cells treated with 50 µM iSGLT2 for 72 h (iSGLT2). Control cells (Ctr) were maintained in complete culture medium with the corresponding volume of HBSS-10 mM Hepes. M = molecular weight markers, lane 1 = Ctr, lane 2 = iSGLT2. Western blotting is expressed as arbitrary units (AU), mRNA levels are reported as floating bars with a line representing the median ± SD of n = 3 independent experiments. †*p* < 0.01 vs. Ctr; ‡*p* < 0.001 vs. Ctr, by unpaired Student’s *t*-test

Evidence on oxidative stress and apoptotic pathway led to further investigate the role of SGLT2 in mitochondrion cytotoxicity by evaluating mitochondrial respiration and glycolytic state (Fig. 4 and Fig. S4 and Fig. S5). In HCT 116 cells, treatment with iSGLT2 decreased the GSH/GSSG ratio (*p* < 0.01), along with ATP-production coupled respiration (*p* < 0.001), maximal and basal respiration (*p* < 0.01), and the percentage of coupling efficiency (*p* < 0.001) (Fig. 4A–E and Fig. S4A, B). Given the major role of SIRT3 in the regulation of cancer metabolism and mitochondrial homeostasis, its levels under iSGLT2 treatment in CRC cells were evaluated [44]. Results showed that the iSGLT2-mediated mitochondrial injury led to the upregulation of SIRT3 activity (*p* < 0.01) as well as mRNA and protein levels (*p* < 0.01) (Fig. 4F–I). These results were confirmed also in HT-29 cell line (Figs. S4 and S5).

Overall, treatment of CRC cells with iSGLT2 decreased GSH/GSSG ratio, impaired mitochondrial respiration and increased SIRT3 protein levels and activity.

### iSGLT2 triggers ER stress and autophagic flux

**Fig. 5:**
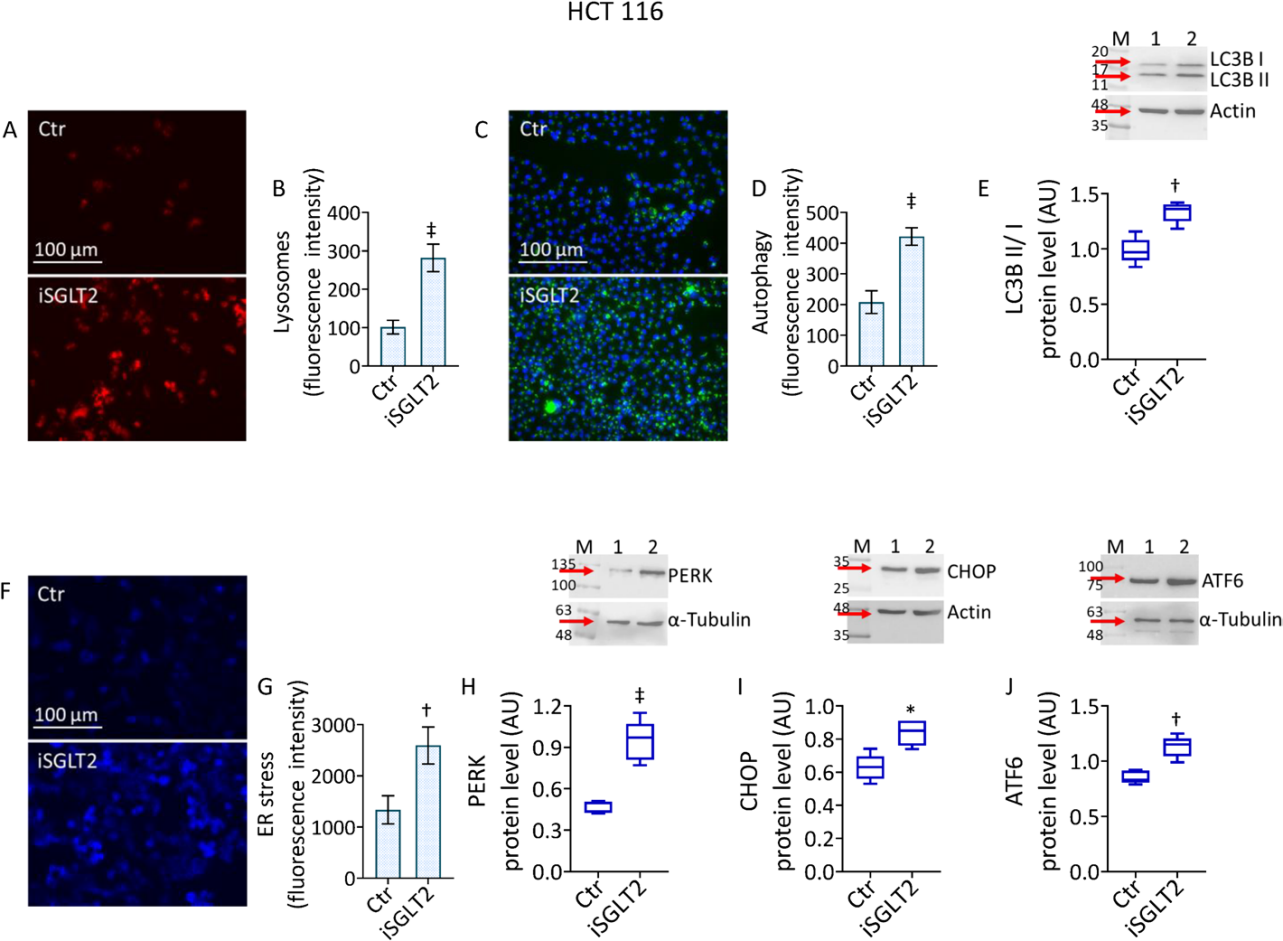
iSGLT2 promotes autophagic flux and ER stress in CRC. Representative fluorescent images and cytofluorimetric detection of (**A**, **B**) lysosomes, (**C**,**D**) autophagy and (**E**) immunoblotting analysis with cropped blots of LC3B II/I ratio in HCT 116 cell lines. Representative fluorescent images and cytofluorimetric detection of (**F**, **G**) ER stress and immunoblotting analysis with cropped blots of (**H**) PERK, (**I**) CHOP and (**J**) ATF6 protein levels in HCT 116 cells treated with 50 µM iSGLT2 for 72 h (iSGLT2). Control cells (Ctr) were maintained in complete culture medium with the corresponding volume of HBSS-10 mM Hepes. Scale bars = 100 µm. M = molecular weight markers, lane 1 = Ctr, lane 2 = iSGLT2. Western blotting results are expressed as arbitrary units (AU). *p < 0.05 vs. Ctr; †*p* < 0.01 vs. Ctr; ‡*p* < 0.001 vs. Ctr, by unpaired Student’s *t*-test

The crosstalk between mitochondrial function and autophagy, both part of the global endoplasmic reticulum (ER) stress response [45], prompted to explore the occurrence of autophagy and ER stress under SGLT2 inhibition (Fig. 5 and Figs. S6, S7). These two phenomena were assessed by evaluating the protein expression levels of autophagy marker, LC3B II/I, and ER stress markers, PERK, CHOP and ATF6. In HCT 116 cells, results showed that iSGLT2 triggered lysosome accumulation (*p* < 0.001) associated to autophagic mechanism (*p* < 0.001) via LC3B II/I upregulation (*p* < 0.01) (Fig. 5A–E and Fig. S6A, B). These phenomena were accompanied by ER stress (*p* < 0.01) sustained by upregulated PERK, CHOP and ATF6 protein levels (*p* < 0.01) (Fig. 5F–J and Fig. S6C). Similarly, in HT-29 cells, iSGLT2 induced lysosome accumulation (*p* < 0.001), LC3B II/I-related autophagy (*p* < 0.001) and ER stress (*p* < 0.01) mediated by increased PERK, CHOP and ATF6 protein expression (*p* < 0.01) (Fig. S6D–F and Fig. S7).

In conclusion, iSGLT2 treatment provoked autophagy via LC3B II/I upregulation, induced ER stress via upregulation of PERK, CHOP and ATF6 protein levels, in CRC cells.

### siSIRT3 opposes iSGLT2-dependent cytotoxicity and mitochondrial injury

**Fig. 6:**
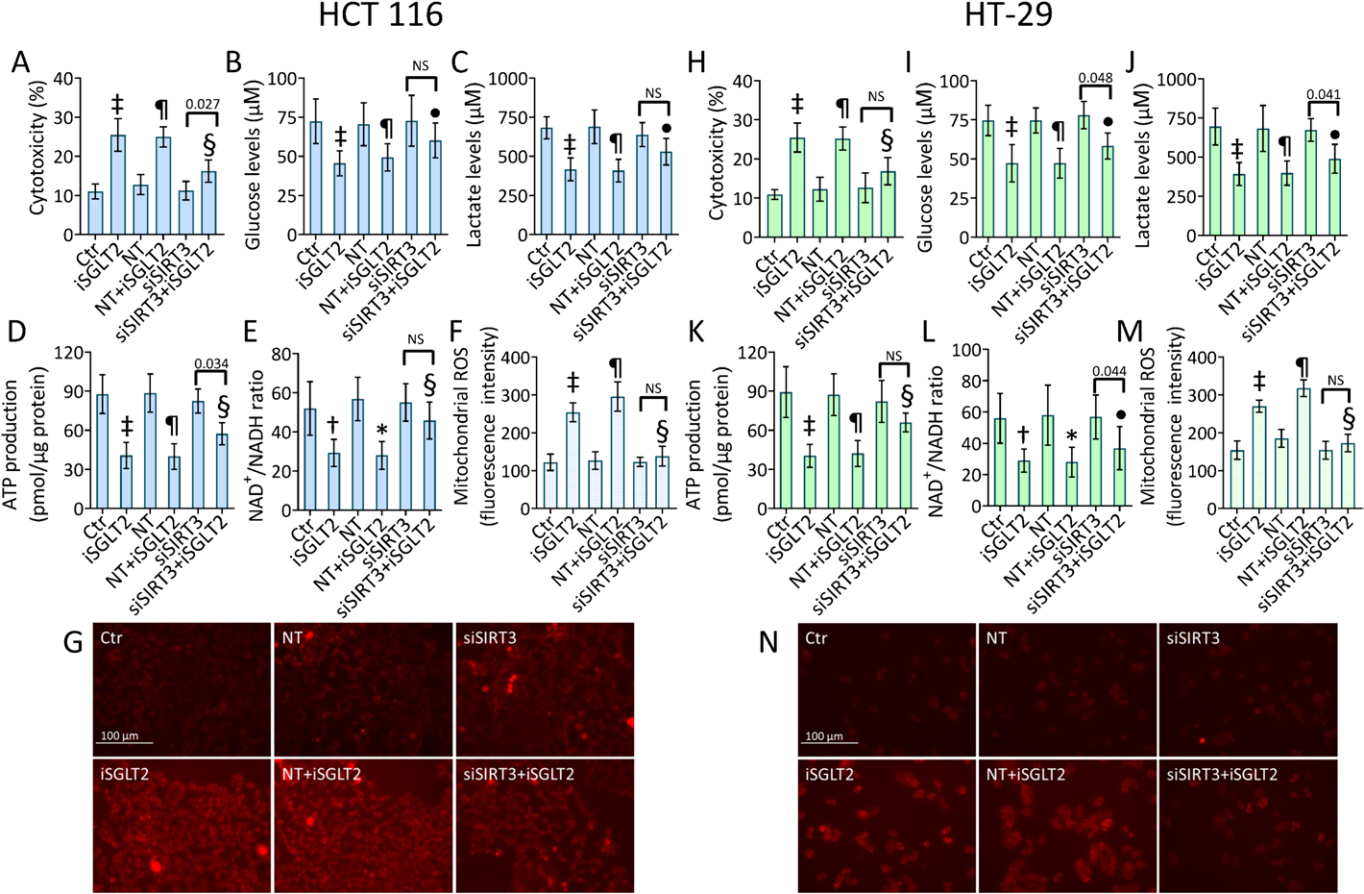
siSIRT3 opposes iSGLT2-dependent cytotoxicity and mitochondrial injury. Detection of (**A**, **H**) cytotoxicity, (**B**, **I**) glucose, (**C**, **J**) lactate (**D**, **K**) ATP and (**E**, **L**) NAD^+^/NADH levels and cytofluorimetric detection and representative fluorescent images of (**F**, **G** and **M**, **N**) mitochondrial ROS in HCT 116 and HT-29 cells treated with 50 µM iSGLT2 for 72 h (iSGLT2) or transfected with nontargeting siRNA control (NT) or SIRT3 siRNA (siSIRT3) before exposure to iSGLT2 (NT + iSGLT2 or siSIRT3 + iSGLT2). Control cells (Ctr) were maintained in complete culture medium with the corresponding volume of HBSS-10 mM Hepes. Scale bars = 100 µm. †*p* < 0.01 vs. Ctr; ‡*p* < 0.001 vs. Ctr; *p < 0.01 vs. NT; ¶*p* < 0.001 vs. NT; •*p* < 0.05 vs. NT + iSGLT2; §*p* < 0.01 vs. NT + iSGLT2. Significance was determined using one-way ANOVA with Tukey post hoc test

Given the results on SIRT3 modulation and taking into account the debatable role played by this sirtuin in CRC carcinogenesis [37, 42, 46–48], the effects of SIRT3 silencing (siSIRT3) during iSGLT2 treatment were explored (Fig. 6 and Fig. S8). Results showed that SIRT3 downregulation opposed the iSGLT2-mediated cytotoxicity but only partially since never reached the control conditions (*p* < 0.01 vs. NT + iSGLT2), as well as the reduction of glucose, lactate, ATP and NAD^+^/NADH content in HCT 116 cells (*p* < 0.01 vs. NT + iSGLT2) (Fig. 6A–E). SIRT3 silenced cells also revealed a decreased mitochondrial ROS amount compared to NT + iSGLT2 treatment (*p* < 0.01) (Fig. 6F, G and Fig. S8). In addition, siSIRT3 alone yielded no different effects compared to the control cells. Similar results were observed in HT-29 cells. Indeed, SIRT3 inhibition attenuated the iSGLT2-related cytotoxicity (*p* < 0.01 vs. NT + iSGLT2), counteracted the impaired glucose, lactate, ATP and NAD^+^/NADH levels (*p* < 0.05 vs. NT + iSGLT2), as mitochondrial ROS generation (*p* < 0.01 vs. NT + iSGLT2) (Fig. 6H–N and Fig. S8). In summary, transient SIRT3 gene silencing opposed iSGLT2-induced cytotoxicity, metabolic and redox alteration in CRC cells.

### siSIRT3 counteracts cell death mechanisms induced by iSGLT2

**Fig. 7:**
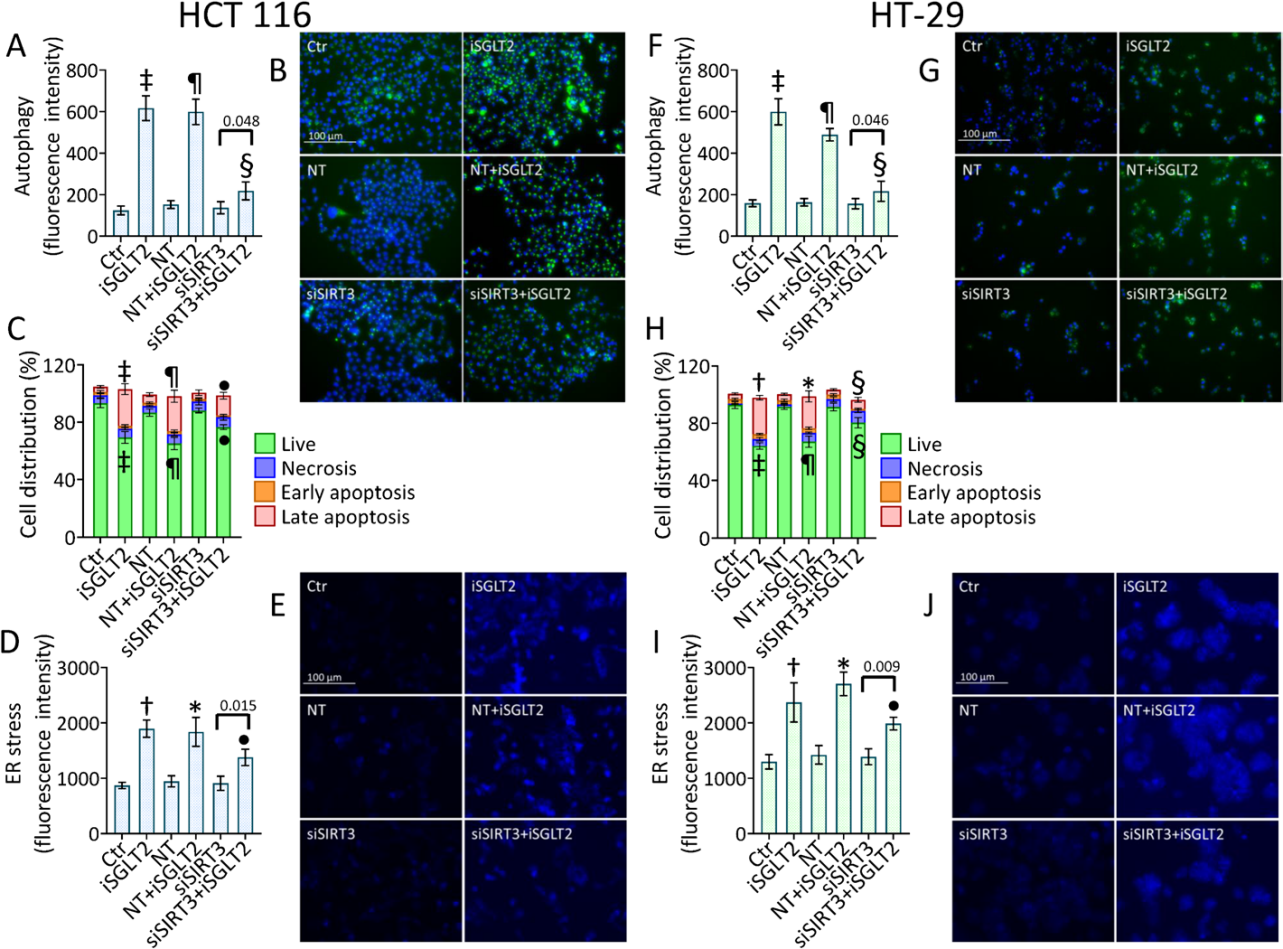
siSIRT3 counteracts cell death mechanisms induced by iSGLT2. Representative cytofluorimetric detection and fluorescent images of (**A**, **B** and **F**, **G**) autophagy, (**C**, **H**) analysis of annexin V-FITC and propidium iodide (PI)-staining and (**D**, **E** and **I**, **J**) ER stress in HCT 116 and HT-29 cells treated with 50 µM iSGLT2 for 72 h (iSGLT2) or transfected with nontargeting siRNA control (NT) or SIRT3 siRNA (siSIRT3) before exposure to iSGLT2 (NT + iSGLT2 or siSIRT3 + iSGLT2). Control cells (Ctr) were maintained in complete culture medium with the corresponding volume of HBSS-10 mM Hepes. Scale bars = 100 µm. †*p* < 0.01 vs. Ctr; ‡*p* < 0.001 vs. Ctr; *p < 0.01 vs. NT; ¶*p* < 0.001 vs. NT; •*p* < 0.05 vs. NT + iSGLT2; §*p* < 0.01 vs. NT + iSGLT2. Significance was determined using one-way ANOVA with Tukey post hoc test

Transient SIRT3 deprivation opposed the iSGLT2-related cell death mechanisms in HCT 116 and HT-29 cells (Fig. 7 and Fig. S9). Results showed attenuated autophagy (*p* < 0.01 vs. NT + iSGLT2), apoptosis (*p* < 0.05 vs. NT + iSGLT2) and ER stress (*p* < 0.01 vs. NT + iSGLT2) when SIRT3 was silenced in HCT 116 cells under iSGLT2 exposure (Fig. 7A–E and Fig. S9A–C). Likewise, in HT-29 results showed decreased rate of autophagy (*p* < 0.01 vs. NT + iSGLT2), apoptotic cell death (*p* < 0.01 vs. NT + iSGLT2) and ER stress (*p* < 0.05 vs. NT + iSGLT2) in siSIRT3 + iSGLT2 cells (Fig. 7F–J and Fig. S9D–F). Overall, silencing of SIRT3 opposed iSGLT2-induced apoptosis, autophagy and ER stress.

### DPP4 as a common target of SGLT2 and SIRT3

**Fig. 8:**
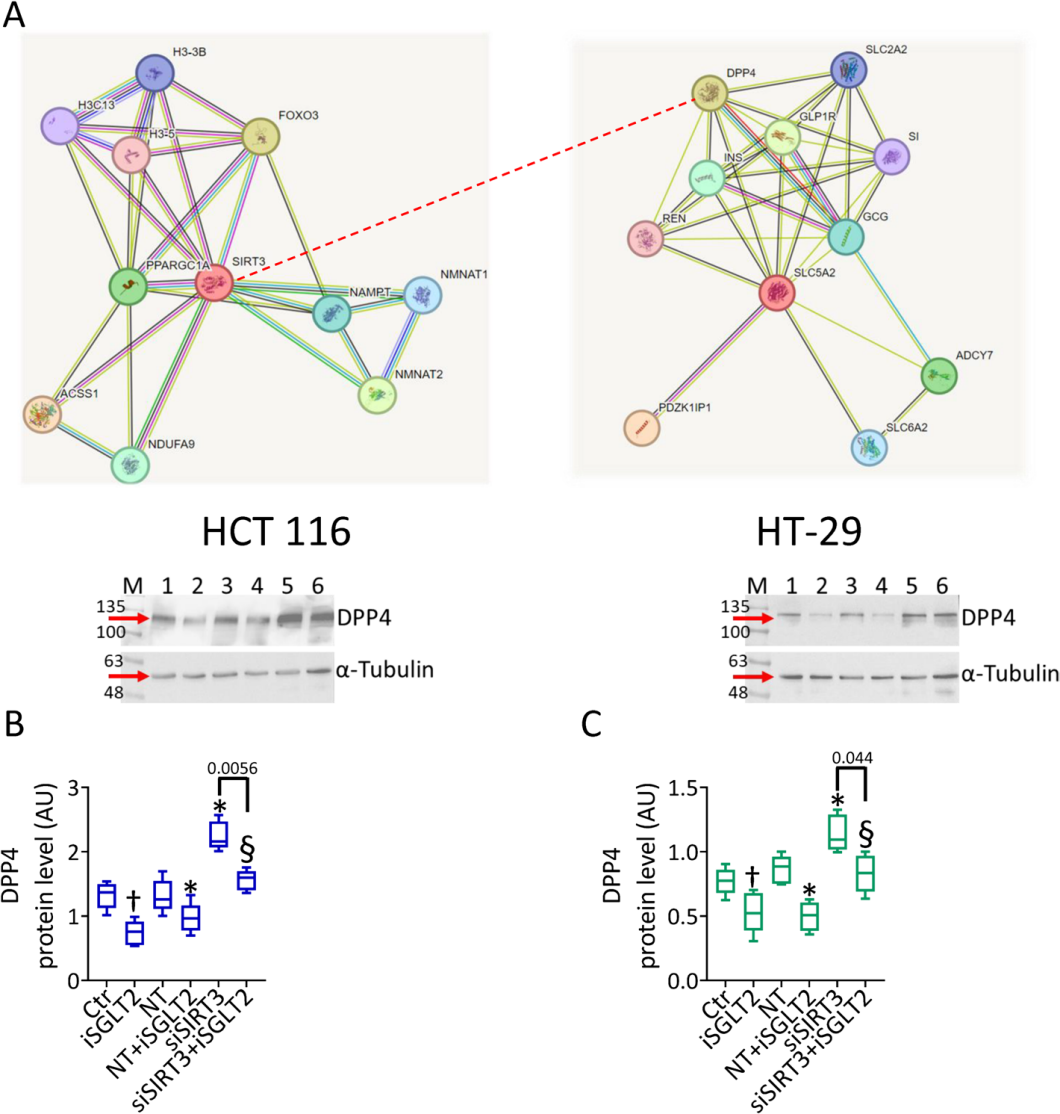
DPP4 as a target of SGLT2 and SIRT3. **A** SIRT3 (left) and SGLT2 (as SLC5A2, right) full interaction networks estimated on STRING database (https://string-db.org/) with the following setting parameters: minimum required interaction score: 0.400; max number of interactors to show in the first shell: 10, and none for the second shell. The dotted line suggests the potential interaction between SIRT3 and DPP4. **B**, **C** Immunoblotting analysis with cropped blots of DPP4 protein levels in HCT 116 and HT-29 cells treated with 50 µM iSGLT2 for 72 h (iSGLT2) or transfected with nontargeting siRNA control (NT) or SIRT3 siRNA (siSIRT3) before exposure to iSGLT2 (NT + iSGLT2 or siSIRT3 + iSGLT2). Control cells (Ctr) were maintained in complete culture medium with the corresponding volume of HBSS-10 mM Hepes. M: molecular weight markers; lane 1: Ctr; lane 2: iSGLT2; lane 3: NT; lane 4: NT + iSGLT2; lane 5: SIRT3^−^; lane 6: siSIRT3 + iSGLT2. †*p* < 0.01 vs. Ctr; *p < 0.01 vs. NT; §*p* < 0.01 vs. NT + iSGLT2. Significance was determined using one-way ANOVA with Tukey post hoc test

The ability of SIRT3 silencing to counteract the iSGLT2-mediated mechanisms, prompted further studies elucidating a possible SGLT2/SIRT3 common target. Analyses by protein–protein interaction databases evidenced dipeptidyl peptidase 4 (DPP4) as direct target of SGLT2 with high confidence score (Fig. 8A), whilst no interaction was found with SIRT3, although recent evidence described a SIRT3-DPP4 interaction [49]. Moreover, recent evidence has shown that DPP4 promotes the development, progression and metastasis of CRC, making it a potential and novel therapeutic target for CRC [50]. To this end, to investigate the SGLT2 and SIRT3 capacity to modulate DPP4 protein levels, immunoblotting analysis with both iSGLT2 and SIRT3 silencing was performed (Fig. 8B,C). Treatment with iSGLT2 led to downregulated DPP4 protein levels (*p* < 0.01 vs. Ctr and NT). SIRT3 deprivation increased the peptidase expression in HCT 116 and HT-29 cells (Fig. 8B,C) (*p* < 0.01 vs. NT). Compared to SIRT3^−^, the siSIRT3 + iSGLT2 slightly decreased DPP4 protein levels only in HT-29 cells (*p* = 0.044), while no difference were observed in HCT 116 cells (*p* = 0.056). Of contrary, the siSIRT3 + iSGLT2 cells showed higher DPP4 protein levels compared to iSGLT2 treatment (*p* < 0.01), suggesting a more pronounced modulating effect exerted by SIRT3 silencing (Fig. 8B,C).

In conclusion, treatment with iSGLT2 induced a downregulation of DPP4 protein levels, whereas silencing of SIRT3 induced an increase in DPP4 protein levels, suggesting a new signaling network in CRC cells.

## Discussion

This study shows first evidence on the role of glucose transporter SGLT2 in CRC cells, as demonstrated by the cytotoxic effect of iSGLT2, canagliflozin, via impaired glucose metabolism and mitochondrial bioenergetic injury. The iSGLT2-mediated metabolic alterations were accompanied by SIRT3 upregulation, cell cycle arrest, apoptotic cell death, autophagy and ER stress occurrence in CRC cells. Moreover, SIRT3 deprivation reduced the iSGLT2-related cytotoxicity, metabolic and mitochondrial damage, and the induction of cell death mechanisms. Analysis by protein–protein interaction database unveiled dipeptidyl peptidase 4 (DPP4) as SGLT2 molecular target. Immunoblotting detection confirmed the DPP4-SGLT2 interplay and, of note, provided the first evidence of a DPP4 modulation exerted by SIRT3.

Aberrant proliferation and aggressiveness of cancer are related to alterations in metabolic parameters. Glucose metabolism usually is deregulated through the different oncogenic stages of CRC progression and is associated to suppression of immune cells [5, 24]. Metabolic reprogramming has been recognized as one of the main mechanisms sustaining CRC initiation and development, thus, it is conceivable that metabolic impairment, negatively impacting tumor onset, might represent a milestone for innovative anticancer strategies [27–29, 37]. It is well-reported that several cancer cells express the glucose transporter SGLT2, being strictly dependent on glucose utilization for growth and proliferation [4, 7]. SGLT2 has been also described as diagnostic marker and therapeutic target in tumor [51], although many mechanistic aspects remain undebated, as its role in CRC development. Consistent with previous data showing upregulated SGLT2 expression in several cancer models and its association to metastasis, drug resistance, poor prognosis and survival, here, we found increased SGLT2 mRNA and protein levels in CRC cells compared to non-tumor cells [3–7]. In this context, accumulating evidence described the in vitro and in vivo anticancer activity of iSGLT2 as well as its synergistic effects when combined with chemotherapy and radiotherapy [5, 52]. In prostate and lung cancer cells iSGLT2 reduced proliferation and clonogenic survival, alone and in combination with docetaxel and ionizing radiation [33]. Treatment with iSGLT2 opposed glucose uptake and cisplatin resistance in hepatoblastoma cells [6], triggered apoptosis and abrogated breast cancer stemness via miR-128-3p overexpression [53, 54]. Canagliflozin re-sensitized hepatocellular carcinoma to cisplatin by inducing ferroptosis [55], regulated glycolysis and glutamine metabolism, inhibited proliferation and clonogenic survival and enhanced radiotherapy efficacy by suppressing mitochondrial oxidative phosphorylation in non-small cell lung cancer [35]. In CRC patients, treatment with iSGLT2 counteracted cell growth, development and adhesion, and displayed synergic interaction with cetuximab [33, 56–59]. Results of this study showed the ability of canagliflozin to decrease CRC cell viability and induce cytotoxicity, with higher effects in HCT 116 and HT-29 cells, consistent with previous in vitro and in vivo data describing the iSGLT2-mediated inhibition of tumor growth and development [33, 56, 58, 59]. It is conceivable to speculate that the lower iSGLT2-related cytotoxicity in SW480 and LoVo cells might reflect the involvement of other SGLT2-independent metabolic targets responsible of tumor development, suggesting different responsiveness among CRC cells.

The iSGLT2 canagliflozin-mediated cytotoxicity has been related to cell cycle arrest in G1 or G1/S phase and apoptotic cell death via caspase-3 activation in hepatocellular carcinoma and lung and thyroid cancer cells [60–62]. Likewise, the results of this study provided the first evidence that iSGLT2 treatment triggered S-phase cell cycle arrest and caspase-3-dependent apoptotic cell death in HCT 116 and HT-29 cells. In line with recent reports, SGLT2 inhibition suppressed glucose uptake, lactate release, ATP production and increased AMPK activation, opposing signaling pathways which contribute to metabolic reprogramming and tumor progression [34, 35, 63]. AMPK is generally phosphorylated to decrease energy consumption and restore energetic metabolism when ATP synthesis is impaired or its consumption is accelerated [64]. Phosphorylated AMPK downregulates many key anabolic enzymes while promoting ATP production, regulating lipid and glucose metabolism, autophagy and inhibiting tumor growth [65]. Rapid activation of AMPK is a cell response to energetic stress and mitochondrial poisons [66]. In this study, we found that iSGLT2 treatment induced oxidative stress, inhibited GSH/GSSG ratio and mitochondrial respiration in CRC HCT 116 and HT-29 cells, as reported in prostate and lung cancer [33–35]. We found that iSGLT2 promoted lysosome accumulation and autophagy cell death via LC3B activation in CRC cells and along with ER stress induction and upregulation of the molecular players related to autophagy triggered by ER stress, namely ER-phagy, i.e., PERK, CHOP and ATF6 [45, 67, 68]. Several reports described the ER stress-mediated autophagy-associated cell death as antitumor strategies in CRC [39, 69–71]. However, to date no studies explored the association between SGLT2 and ER stress/autophagy as antineoplastic tool in CRC. In HCT 116 and HT-29 the iSGLT2-induced ER-phagy cell death mechanism was also accompanied by SIRT3 upregulation. Increasing evidence closely relate SIRT3 to clinical outcomes of CRC, acting as a tumor promoter or suppressor [40, 72–74].

In cancer, SIRT3 targets different enzymes regulating oxidative stress and cell metabolism [75, 76] thus acting as a tumoral promoter, by maintaining ROS content under specific thresholds, or tumor suppressor by triggering cell death under stress conditions [77]. SIRT3 is highly expressed in CRC cells leading to tumor resistance and poor overall survival [78, 79], with the reduction of protein expression resulting in cytotoxicity, cell death and impaired metabolic state and aggressiveness [42, 74]. Of contrary, other studies described a low expression of SIRT3 as predicting poor prognosis in CRC patients and its upregulation related to metabolic reprogramming and cell death mechanisms in in vitro models [37, 46, 47]. Increased SIRT3 activity and expression resulted in altered mitochondrial activity via ROS generation, loss of mitochondrial membrane potential, metabolic reprogramming, apoptosis and necroptotic cell deaths in CRC cells [37, 46, 80]. Studies showed the capacity of empagliflozin to protect heart from doxorubicin cardiomyopathy in mice by enhancing Beclin-1/ toll-like receptor (TLR)9/ SIRT3 axis and the effects of empagliflozin and canagliflozin to ameliorate renal injury by rescuing SIRT3 expression and limiting aberrant glycolysis, oxidative stress, and epithelial-mesenchymal transition (EMT) [36, 81, 82]. In accordance, results of this study showed that treatment with canagliflozin upregulated SIRT3 mRNA and protein levels, and increased content and activity of SIRT3 in HCT 116 and HT-29 cells. At molecular level, transient silencing experiments revealed that SIRT3 deficiency opposed the canagliflozin-mediated cytotoxicity, metabolic alterations and cell death mechanisms, thus reporting the first evidence of SGLT2/SIRT3 interaction in CRC.

Further investigations suggested DPP4 as a downstream target of SGLT2 and SIRT3, as revealed by the ability of both SGLT2 inhibition and SIRT3 silencing to modulate the peptidase protein levels in HCT 116 and HT-29 cells. Recent evidence described the crucial role of DPP4 in CRC initiation and progression and demonstrated the association of DPP4-inhibitor treatment with a better prognosis of CRC patients [50, 83]. The correlation of SGLT2/SIRT3 with DDP4 regulation has been independently described, although in different experimental models [49, 84]. Treatment with the iSGLT2 dapagliflozin reduced DPP4 serum levels in patients with type 2 diabetes and non-alcoholic fatty liver disease [85], while the DPP4 inhibitor sitagliptin ameliorated the ischemia/reperfusion-induced injury in cardiomyocytes by mediating SIRT3 upregulation and autophagy [49]. In accordance, our results revealed a negative DPP4 regulation under iSGLT2 exposure, with a significant peptidase overexpression when SIRT3 was silenced, suggesting DPP4 as a novel molecular target of SGLT2 and unveiling its value as therapeutic tool in CRC.

Despite significant improvements in diagnosis and therapeutic approaches, CRC management is not yet at a satisfactory level since the mortality rate increases globally over the years and a better understanding of underlying molecular mechanisms is needed to find new effective agents for CRC treatment [85]. Screening programs and novel surgical techniques allowed a reduction of CRC mortality rates in non-metastatic patients, although about 22% of CRC-related deaths have metastasis which cannot be surgically treated and therefore treatment with conventional chemotherapies represents the only option [86, 87]. To date, metastasis is the leading mortality cause in CRC patients [25, 26, 88]. In this context, search of new targets for precision therapeutics and innovative agents to overcome traditional medicine limits are compelling necessities. The present study unveils a crucial role of iSGLT2 in glucose metabolism and metabolic energetic reprogramming in CRC tumorigenesis, suggesting a dynamic interplay among metabolic alteration, damaged mitochondrial bioenergetic machinery, and the induction of programmed cell death mechanisms in response to SGLT2 suppression. A mechanistic approach unveiled the SGLT2-SIRT3 interaction and deepened molecular targets involved in SGLT2-mediated antineoplastic effects.

## Conclusions

Altogether, these findings, adding knowledge on SGLT2 role in cancer development and progression, support its potential as crucial target in CRC treatment. Undoubtedly, other roles of SGLT2 in the development and prognosis of CRC warrant further investigation. It is undeniable that other regulators and pathways may be involved in SGLT2-mediated cytotoxicity. Further studies to pinpoint additional processes through which SGLT2 modulates CRC viability and to better understand how SGLT2 affects SIRT3 activity and levels are compelling. In addition, validating results in preclinical tumor models are required to overcome differences due to different susceptibilities proper of each cellular model used for the study. Indeed, the mechanisms provided by this study will be of great interest in the field, hopefully providing bases to new future clinical applications of iSGLT2. Nonetheless, important issues still need to be clarified before hypothesizing an in human application. Firstly, the effects evaluated in vitro are the result of cells exposure to high drug dosages, which would imply high drug site-specific accumulation in vivo to obtain the same effect. Also, the in vitro drug concentrations are often higher than the maximum drug blood serum concentration reached in patients with diabetes and heart failure, thus not guaranteeing the safety of the treatment. In addition, the effects evaluated in vitro do not consider the interaction of the drug with other elements of tumor microenvironment, which could result in drug effect modifications. The use of other iSGLT2 agents and their combination with therapy currently employed for CRC treatment, as radiotherapy and chemotherapy, are also to be taken into account in future investigations. Similarly, the role of DPP4 should be validated by further experimental approaches, including treatment with DDP4 inhibitors or knockout models.

### Supplementary Information

Supplementary Material 1.

## Data Availability

The data that support the findings of this study are available in the body of the text and supplemental materials. Raw data of this study are available at the corresponding author upon request.

## Abbreviations

AMPK: AMP-activated protein kinase
ATF6: Activating transcription factor 6
CHOP: C/EBP homologous protein
CRC: Colorectal cancer
Ctr: Control cells
DPP4: Dipeptidyl peptidase 4
ECAR: Extracellular acidification rate
EMT: Epithelial-mesenchymal transition
ER: Endoplasmic reticulum
HBSS: Hanks’ balanced salt solution
iSGLT2: Sodium-glucose transporter 2 inhibitors
LC3B: Microtubule-associated protein 1 light chain 3B
NAM: Nicotinamide
NT: Control non-targeting siRNA
OCR: Oxygen consumption rate
PERK: Protein kinase RNA-like ER kinase
PI: Propidium iodide
ROS: Reactive oxygen species
SDS-PAGE: Sodium dodecyl sulfate–polyacrylamide gel electrophoresis
SGLT2: Sodium-glucose transporter 2
SIRT3: Sirtuin 3
siSIRT3: SIRT3 siRNA
TLR: Toll-like receptor
