# Supplementary material for: SGLT2 inhibitor promotes mitochondrial dysfunction and ER-phagy in colorectal cancer cells
Source: Cell Mol Biol Lett. 2024 May 29;29:80. doi: 10.1186/s11658-024-00599-1 (PMC11134909; doi:10.1186/s11658-024-00599-1)
Supplement: Supplementary file 1 — Supplementary Material 1. [file 11658_2024_599_MOESM1_ESM.docx]

SGLT2 inhibitor promotes mitochondrial dysfunction and ER-phagy in colorectal cancer cells

Camilla Anastasio^1^, Isabella Donisi^1^, Vitale Del Vecchio^2^, Antonino Colloca^1^, Luigi Mele^2^, Celestino Sardu^3^, Raffaele Marfella^3^, Maria Luisa Balestrieri^1^, Nunzia D’Onofrio^1*^

^1^ Department of Precision Medicine, University of Campania Luigi Vanvitelli, 80138 Naples, Italy.

^2^ Department of Experimental Medicine, University of Campania Luigi Vanvitelli, Via Luciano Armanni 5, 80138 Naples, Italy.

^3^ Department of Advanced Clinical and Surgical Sciences, University of Campania Luigi Vanvitelli, 80138 Naples, Italy.

* Correspondence to Nunzia D’Onofrio, nunzia.donofrio@unicampania.it

SUPPLEMENTARY MATERIAL

**
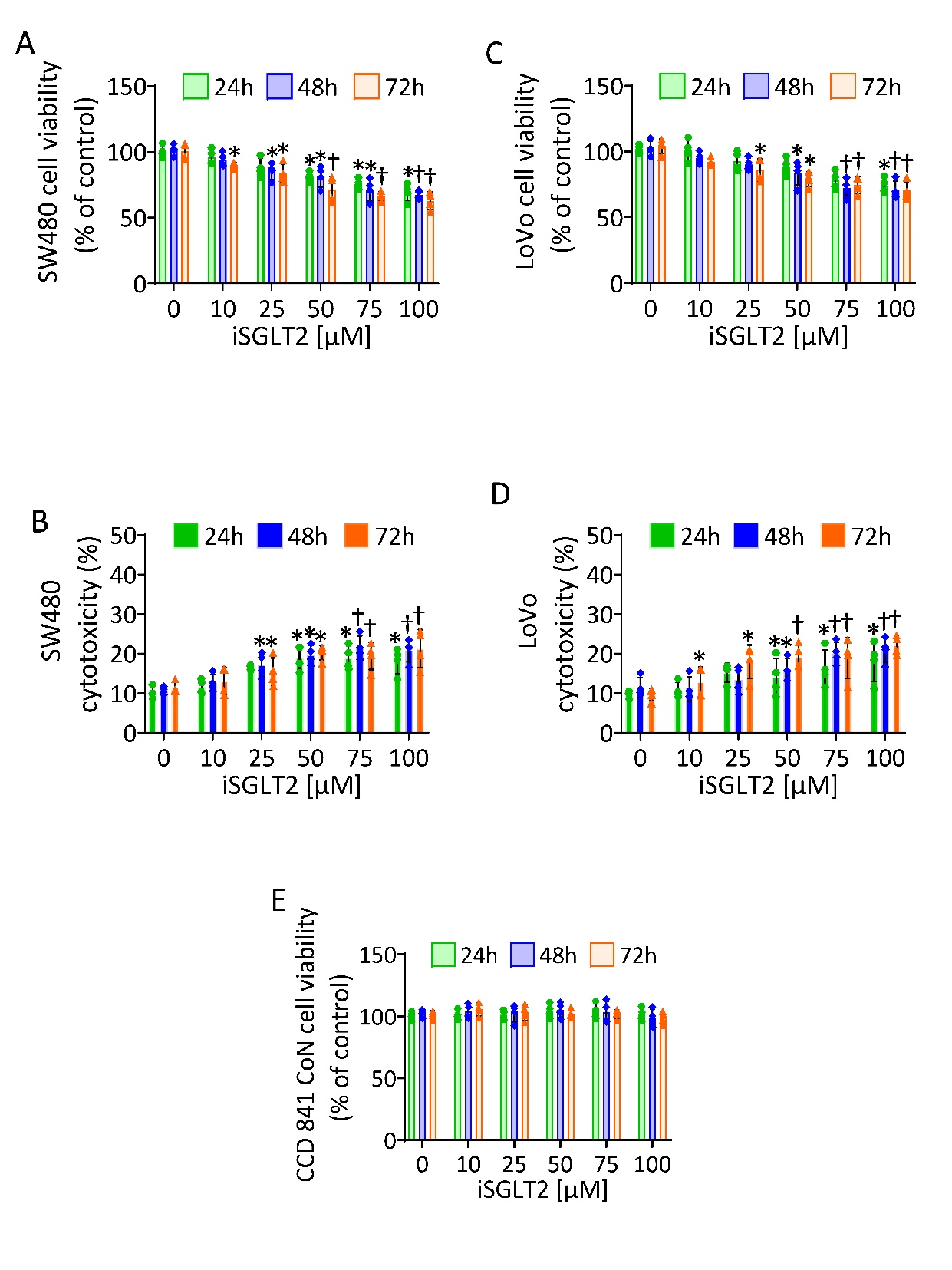
**

**Supplementary Figure S1.** iSGLT2 treatments. Colorectal cancer cell viability and cytotoxicity evaluated in (A,B) SW480 and (C,D) LoVo treated with iSGLT2 (0-100 µM) for 24, 48 and 72h. Control cells (0 µM) were maintained in complete culture medium with the corresponding highest volume of HBSS-10 mM Hepes. *p<0.05 vs. 0 µM; †p<0.01 vs. 0 µM. (E) Cell viability assessed in non-tumor CCD 841 CoN cells treated for 24, 48 and 72h with increasing concentrations of iSGLT2 (0-100 µM).


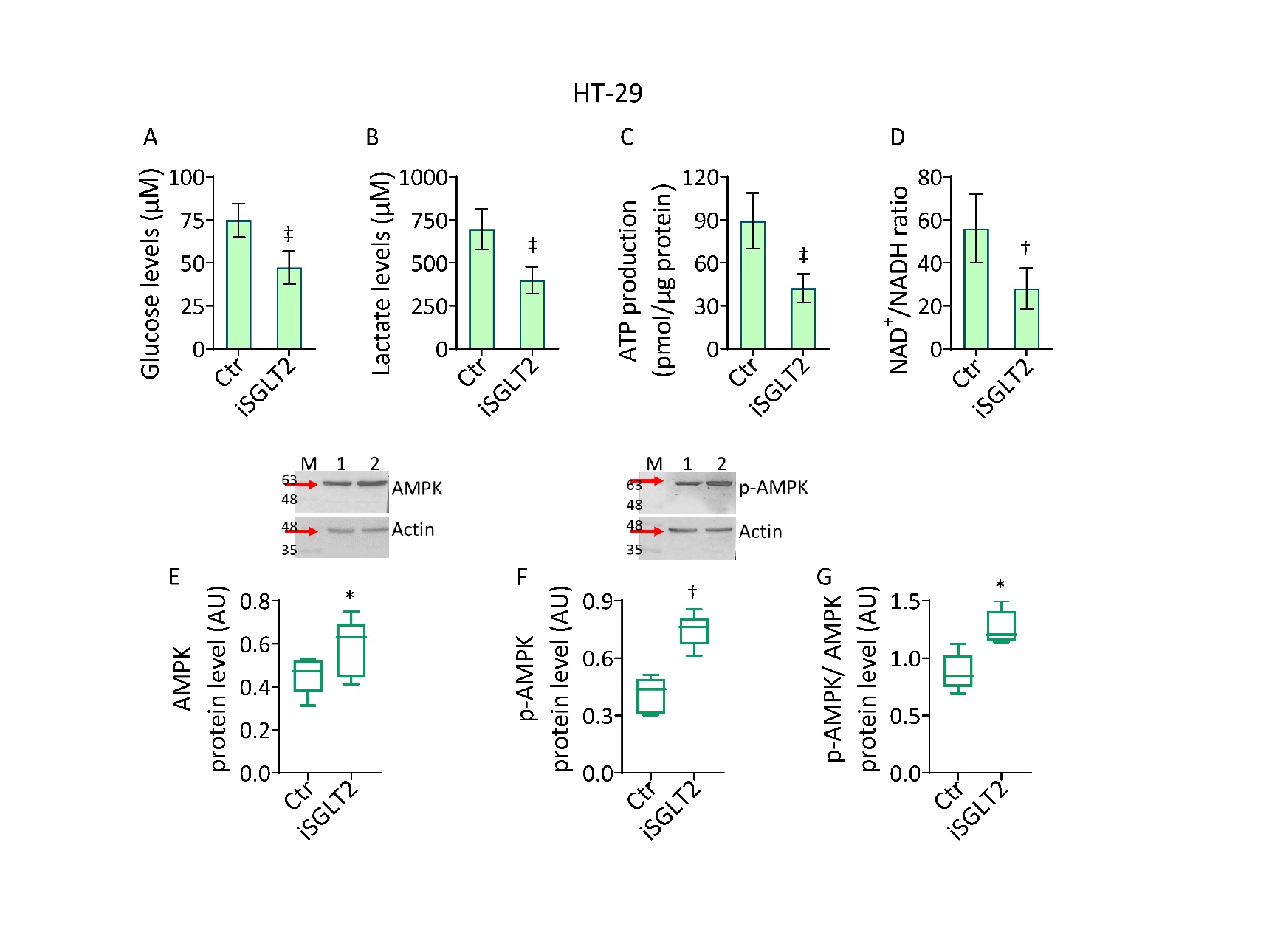


**Supplementary Figure S2.** iSGLT2 affects glucose metabolism. Evaluation of (A) glucose, (B) lactate, (C) ATP and (D) NAD^+^/NADH levels and immunoblotting analysis with cropped blots of (E) AMPK, (F) p-AMPK and (G) p-AMPK/AMPK ratio in HT-29 cells treated with 50 µM iSGLT2 for 72h (iSGLT2). Control cells (Ctr) were maintained in complete culture medium with the corresponding volume of HBSS-10 mM Hepes. M = molecular weight markers, lane 1 = Ctr, lane 2 = iSGLT2. Western blotting results are expressed as arbitrary units (AU). *p<0.05 vs. Ctr; †p<0.01 vs. Ctr; ‡p<0.001 vs. Ctr, by unpaired Student’s t- test.

**
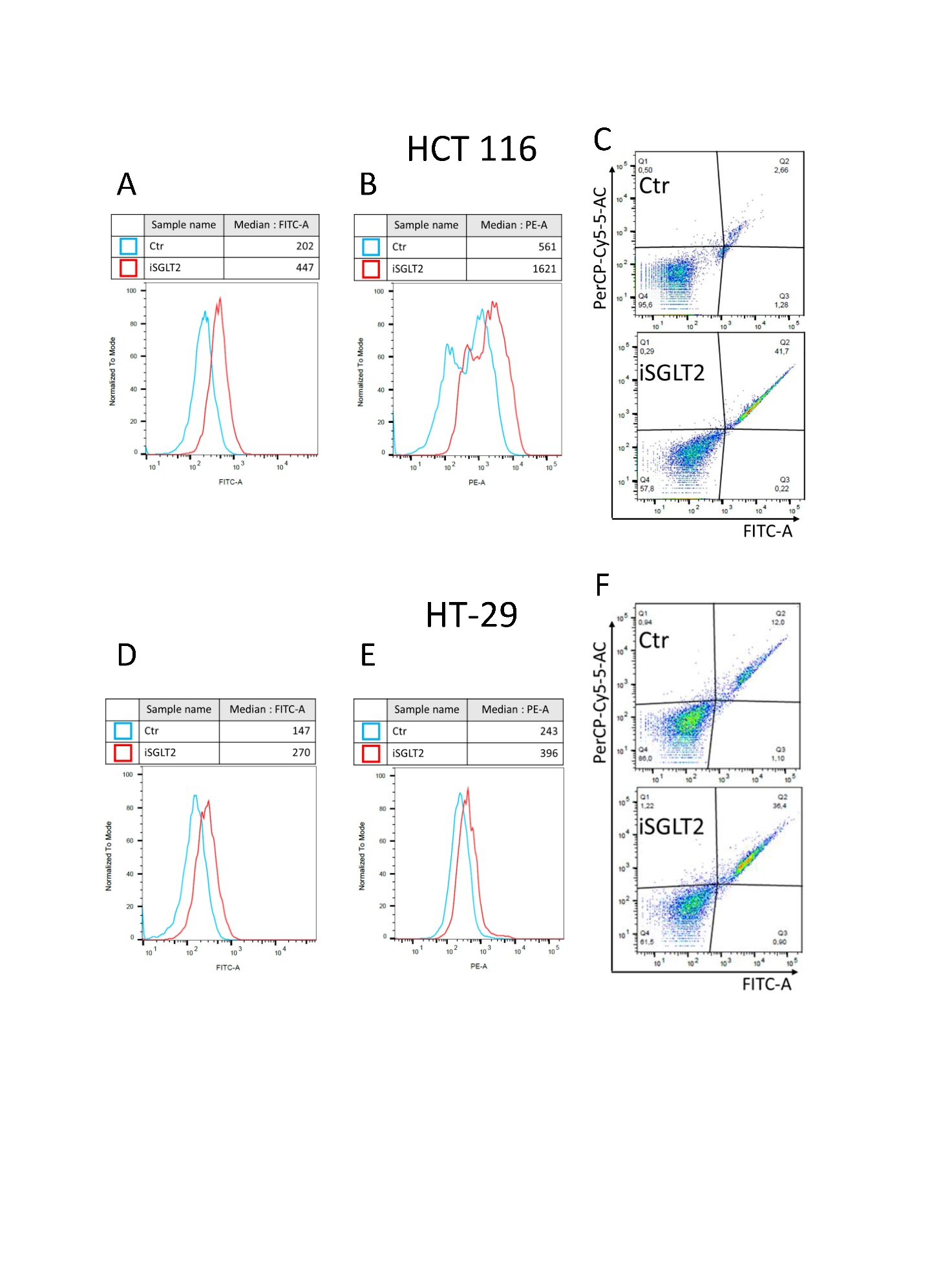
**

**Supplementary Figure S3.** Oxidative state and apoptotic cell death analyses. Representative FACS analysis of (A,D) intracellular and (B,E) mitochondrial ROS and dot plots of (C,F) caspase-3/7 activation in HCT 116 and HT-29 cells treated with 50 µM iSGLT2 for 72h (iSGLT2). Control cells (Ctr) were maintained in complete culture medium with the corresponding volume of HBSS-10 mM Hepes.

**
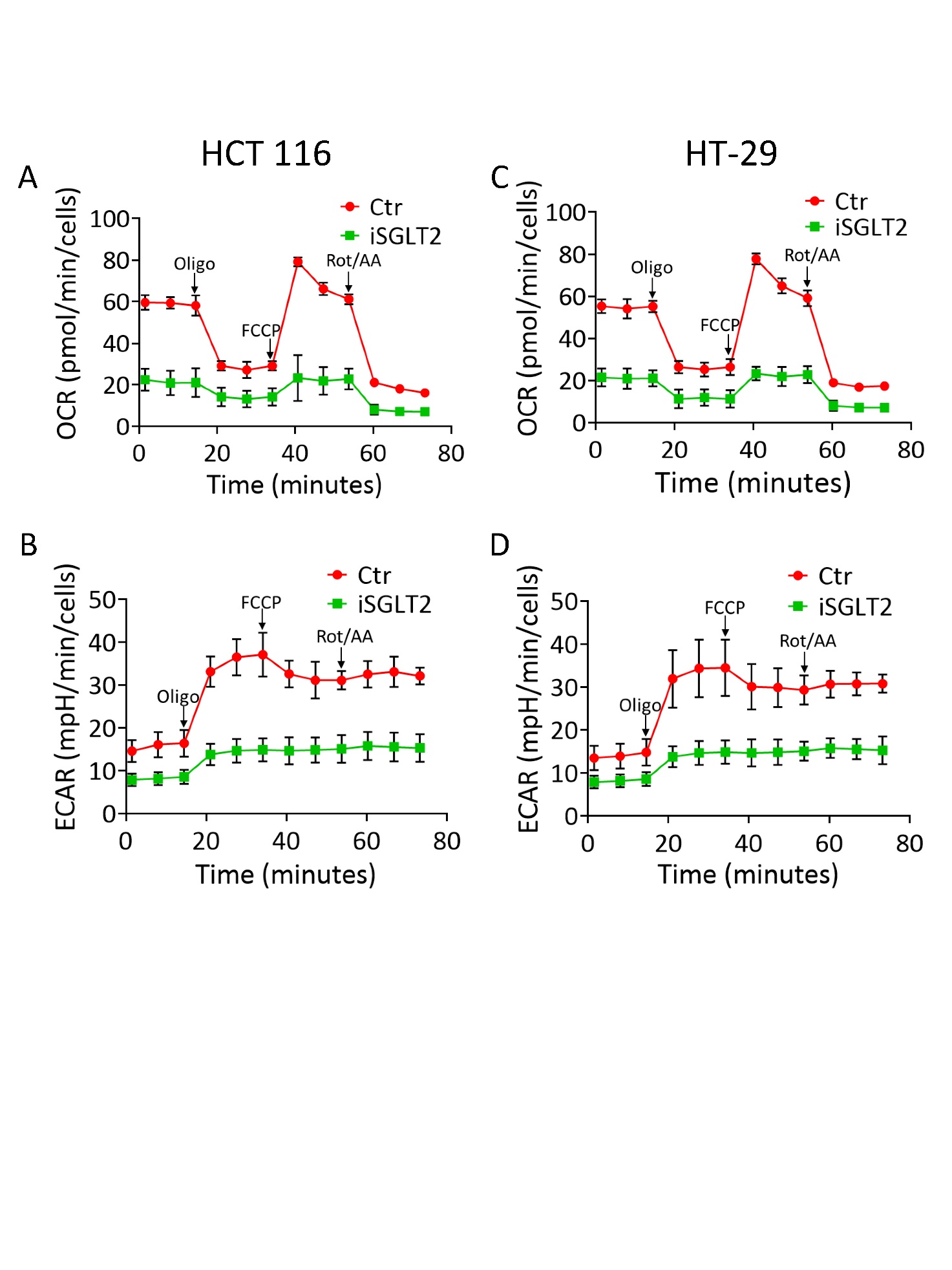
**

**Supplementary Figure S4.** iSGLT2 effects on mitochondrial respiration. (A,C) Oxygen consumption rate (OCR) and (B,D) extracellular acetylation rate (ECAR) assessed with Seahorse analyzer in HCT 116 and HT-29 cells treated with iSGLT2 (iSGLT2) or maintained in complete culture medium with the corresponding volume of HBSS-10 mM Hepes (Ctr).


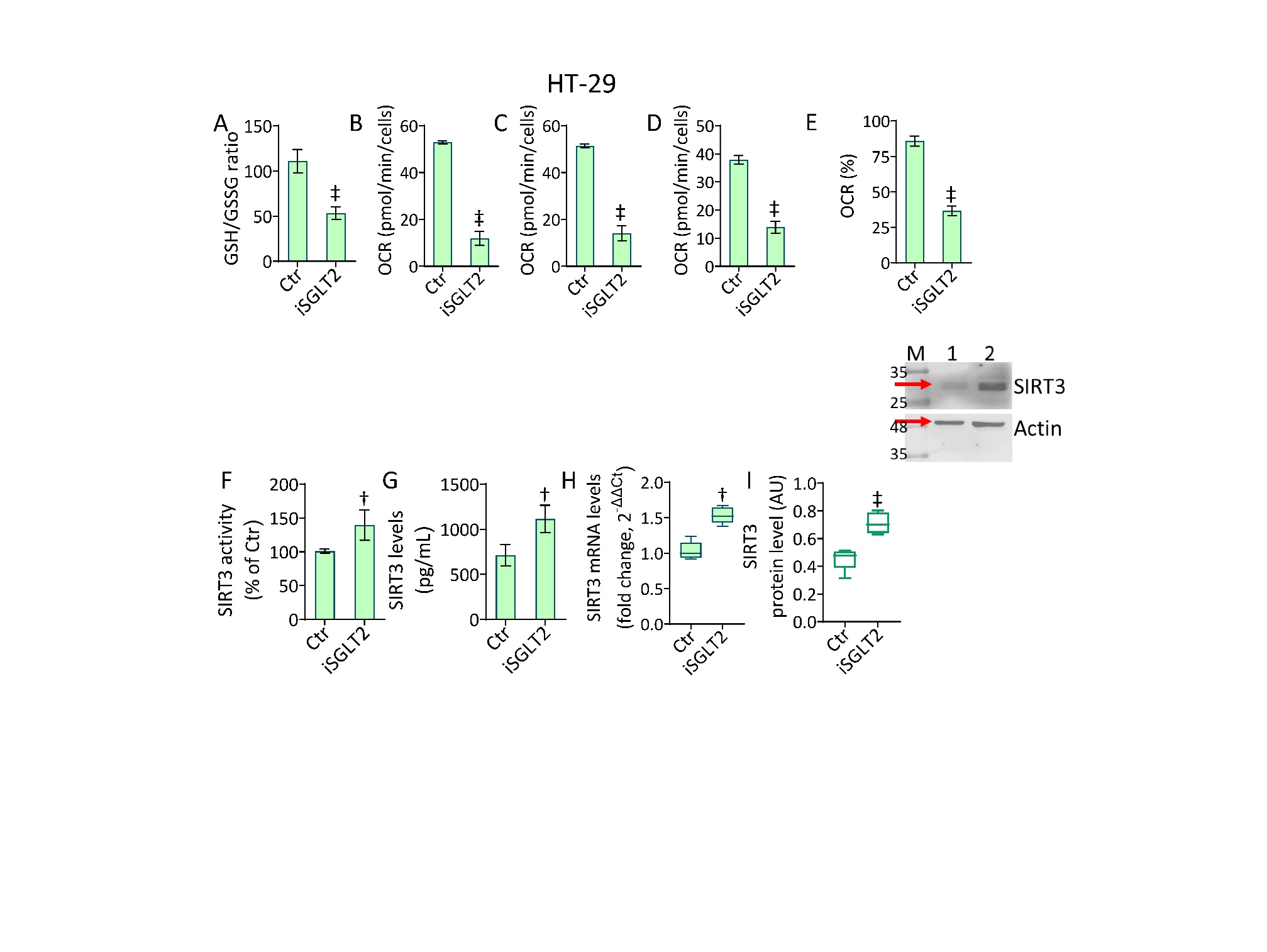


**Supplementary Figure S5.** iSGLT2 impairs energetic state and modulates SIRT3 levels. Evaluation of (A) GSH/GSSG ratio and (B) ATP production coupled respiration, (C) maximal and (D) basal respiration, and (E) coupling efficiency assessed by Seahorse analyzer and (F) SIRT3 activity, (G) content evaluated by ELISA kit, (H) mRNA expression and (I) immunoblotting analysis with cropped blots in HT-29 cells treated with 50 µM iSGLT2 for 72h (iSGLT2). Control cells (Ctr) were maintained in complete culture medium with the corresponding volume of HBSS-10 mM Hepes. M = molecular weight markers, lane 1 = Ctr, lane 2 = iSGLT2. Western blotting is expressed as arbitrary units (AU), mRNA levels are reported as floating bars with a line representing the median ± SD of n = 3 independent experiments. †p<0.01 vs. Ctr; ‡p<0.001 vs. Ctr, by unpaired Student’s t- test.


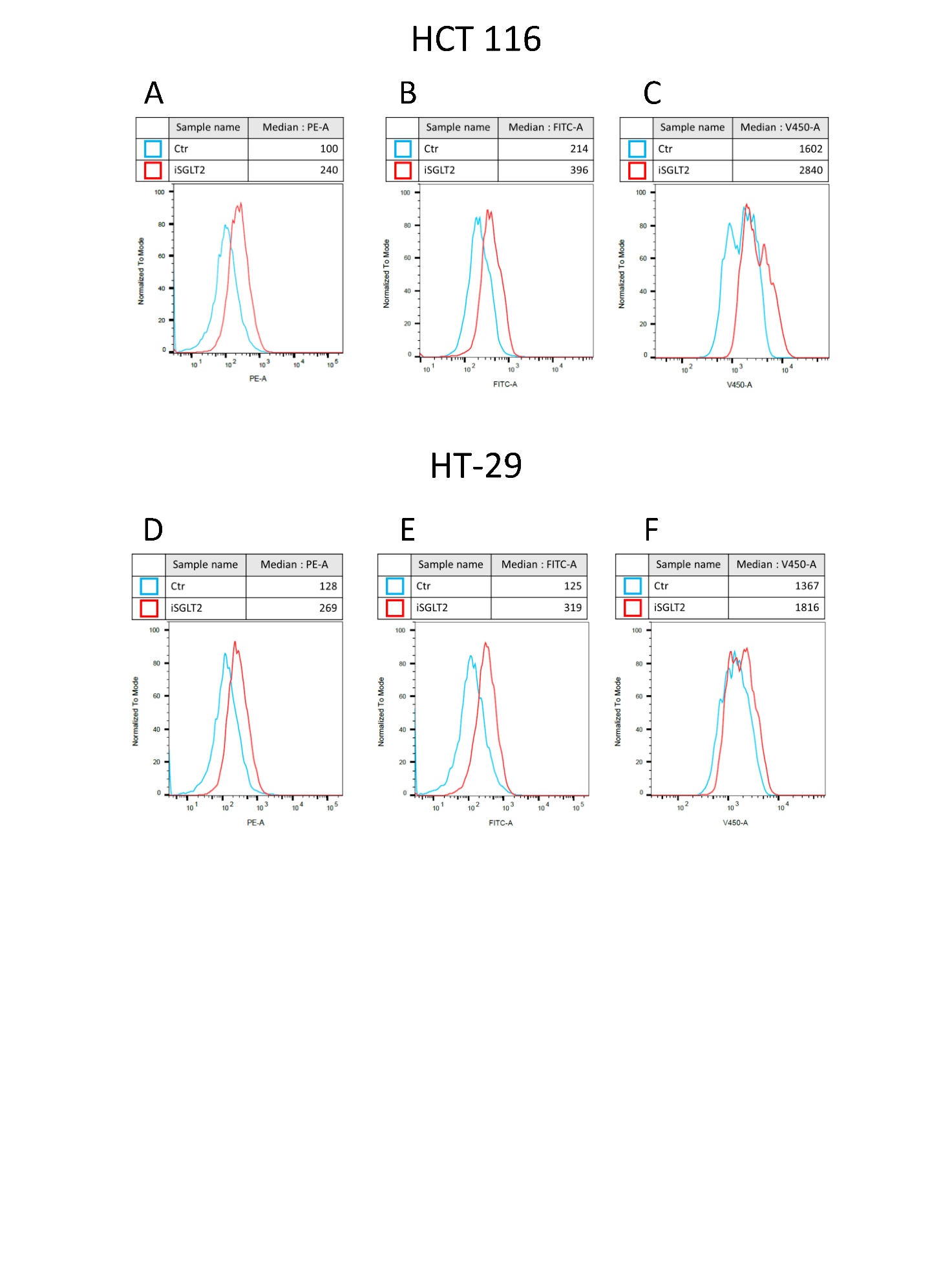


**Supplementary Figure S6.** ER-phagy analysis. Representative FACS analysis of (A,D) lysosomes, (B,E) autophagy and (C,F) ER stress in HCT 116 and HT-29 cells treated with 50 µM iSGLT2 for 72h (iSGLT2). Control cells (Ctr) were maintained in complete culture medium with the corresponding volume of HBSS-10 mM Hepes.


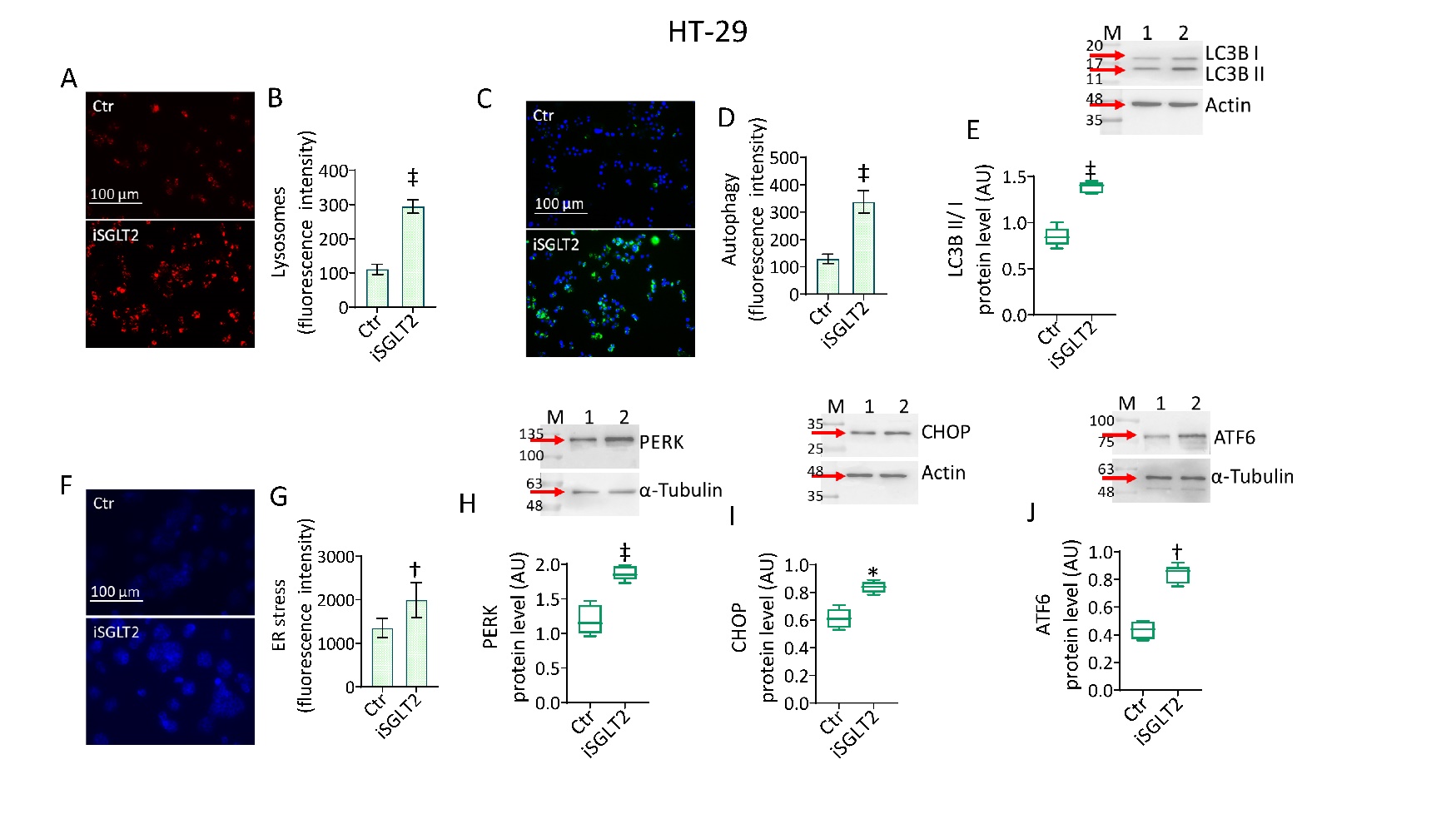


**Supplementary Figure S7.** iSGLT2 promotes autophagic flux and ER stress. Representative fluorescent images and cytofluorimetric detection of (A,B) lysosomes, (C,D) autophagy and (E) immunoblotting analysis with cropped blots of LC3B II/I ratio in HT-29 cell lines. Representative fluorescent images and cytofluorimetric detection of (F,G) ER stress and immunoblotting analysis with cropped blots of (H) PERK, (I) CHOP and (J) ATF6 protein levels in HT-29 cells treated with 50 µM iSGLT2 for 72h (iSGLT2). Control cells (Ctr) were maintained in complete culture medium with the corresponding volume of HBSS-10 mM Hepes. Scale bars = 100 µm. M = molecular weight markers, lane 1 = Ctr, lane 2 = iSGLT2. Western blotting results are expressed as arbitrary units (AU). *p<0.05 vs. Ctr; †p<0.01 vs. Ctr; ‡p<0.001 vs. Ctr, by unpaired Student’s t- test.


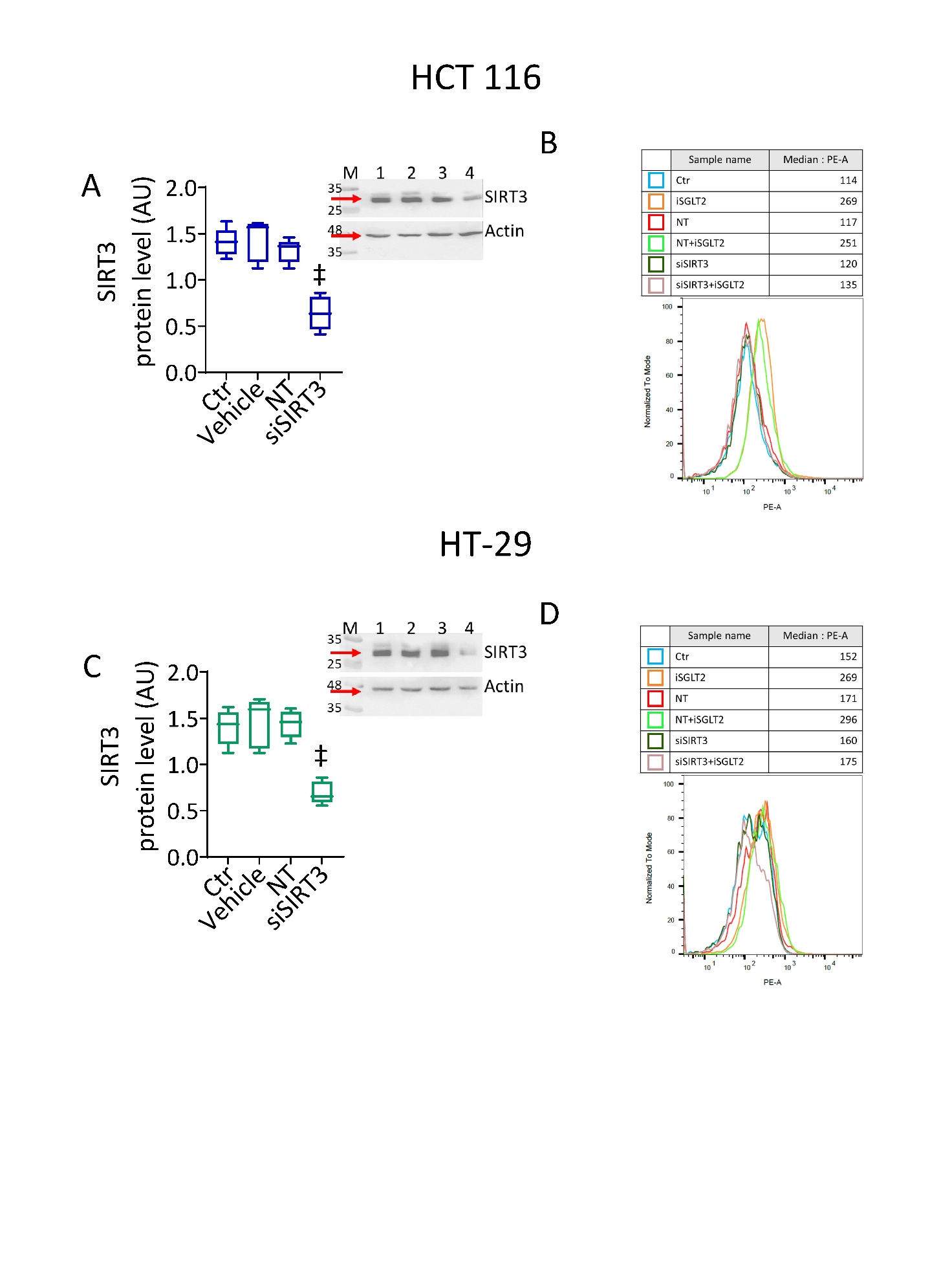


**Supplementary Figure S8.** SIRT3 silencing. (A,C) Representative immunoblotting analysis with cropped blot of SIRT3 protein levels in HCT 116 and HT-29 cells treated with empty transfectant reagent (Vehicle) or transfected with nontargeting siRNA control (NT) or SIRT3 siRNA (siSIRT3). M = molecular weight markers, lane 1 = Ctr, lane 2 = Vehicle, lane 3 = NT, lane 4 = siSIRT3. Western blotting data are expressed as arbitrary units (AU). ‡p<0.001 vs. NT. Representative FACS analysis of (B,D) mitochondrial ROS levels in HCT 116 and HT-29 cells treated with 50 µM iSGLT2 for 72h (iSGLT2) or transfected with NT or siSIRT3 before exposure to iSGLT2 (NT+iSGLT2 or siSIRT3+iSGLT2). Control cells (Ctr) were maintained in complete culture medium with the corresponding volume of HBSS-10 mM Hepes.


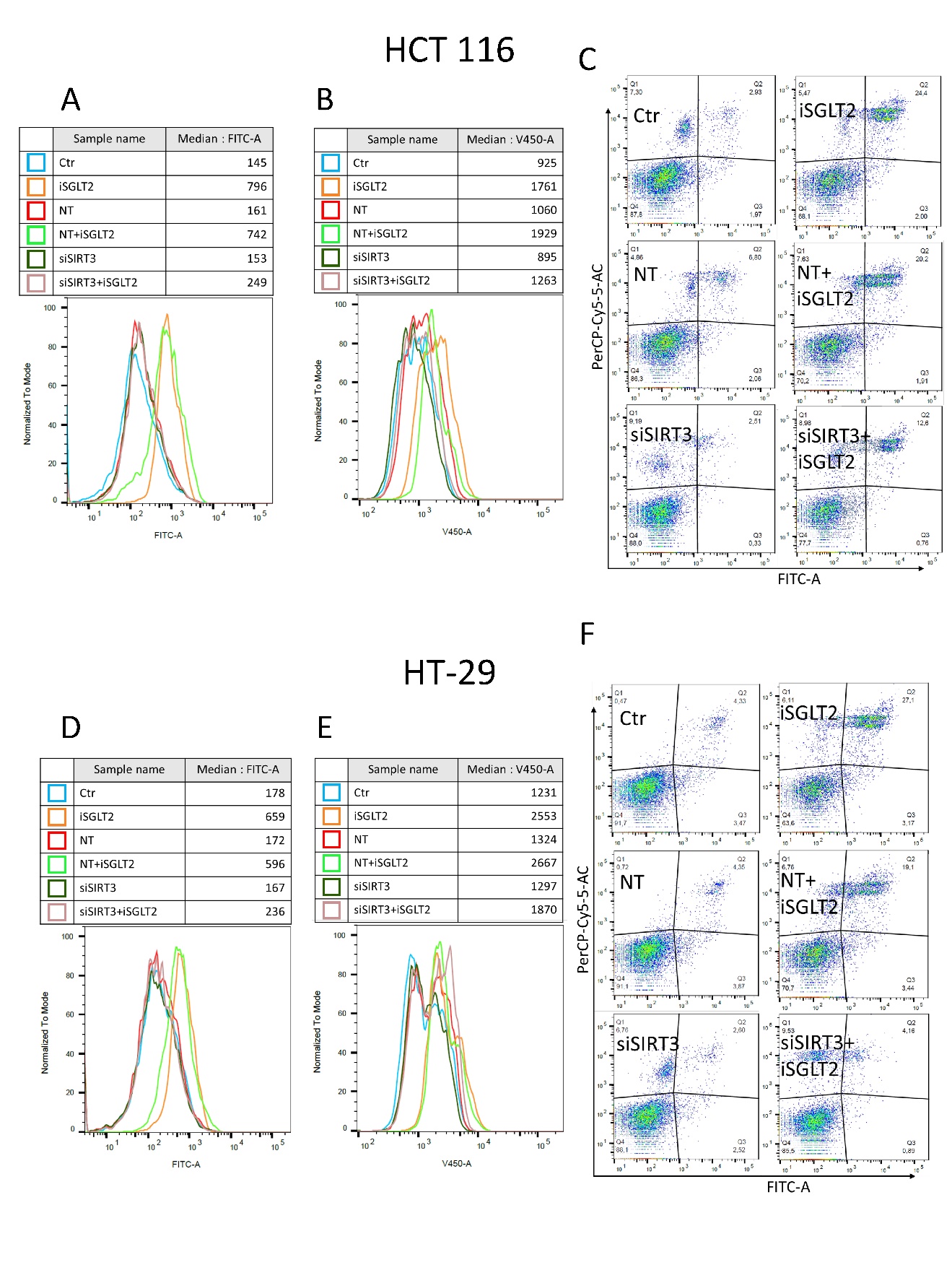


**Supplementary Figure S9.** siSIRT3 effects in programmed mechanisms. Representative FACS analysis of (A,D) autophagy and (B,E) ER stress and (C,F) dot plots of annexin V-FITC and propidium iodide (PI)-staining in HCT 116 and HT-29 cells treated with 50 µM iSGLT2 for 72h (iSGLT2) or transfected with nontargeting siRNA control (NT) or SIRT3 siRNA (siSIRT3) before exposure to iSGLT2 (NT+iSGLT2 or siSIRT3+iSGLT2). Control cells (Ctr) were maintained in complete culture medium with the corresponding volume of HBSS-10 mM Hepes. Lower left quadrant: viable cells; upper left quadrant: necrotic cells; lower right quadrant: early apoptotic cells; upper right quadrant: late apoptotic cells.

**Supplementary Table S1. Primer sequences used in the study.**

| **Gene** | **Forward primer** | **Reverse primer** |
| --- | --- | --- |
| SGLT2 (NM_003041) | F-5’-TCTACTTCGCCATTGTGCTG-3’ | R-5’-ATCTCCATGGCACTCTCTGG-3’ |
| SIRT3 (23410) | F-5’-AGAAGAGATGCGGGACCTTG-3’ | R-5’-GGTCCATCAAGCCTAGAGCAG-3’ |
| GAPDH (2597) | F-5’-GAAGGTGAAGGTCGGAGTC-3’ | R-5’-GAAGATGGTGATGGGATTTC-3’ |
